# Comparisons of the Effectiveness and Safety of Tuina, Acupuncture, Traction, and Chinese Herbs for Lumbar Disc Herniation: A Systematic Review and Network Meta-Analysis

**DOI:** 10.1155/2019/6821310

**Published:** 2019-03-20

**Authors:** Zhuomao Mo, Dong Li, Renwen Zhang, Minmin Chang, Binbin Yang, Shujie Tang

**Affiliations:** School of Chinese Medicine, Jinan University, Guangzhou, Guangdong Province 510632, China

## Abstract

**Background:**

Tuina, acupuncture, traction, and Chinese herbs play an important role in the treatment of lumbar disc herniation. However, the comparative effectiveness and safety of the four commonly utilized treatment modalities are still unclear.

**Objective:**

To compare the effectiveness and safety of the four interventions for lumbar disc herniation.

**Methods:**

Randomized controlled trials comparing any two of the four interventions in the treatment of lumbar disc herniation were identified using the following databases: PubMed, the Cochrane Library, Embase, Web of Science, the China National Knowledge Infrastructure, Chinese Science and Technology Periodical Database, and Wanfang data, and network meta-analysis was performed using STATA 14.0.

**Results:**

One hundred and twenty-one studies involving a total of 13075 patients were included. In all the outcome measurements, traction demonstrated a worst effectiveness, and Tuina and acupuncture demonstrated a best effectiveness, but no significant differences were found between Tuina and acupuncture. Compared with Tuina or acupuncture, Chinese herbs showed a similar effectiveness in Visual Analogue Score and Japanese Orthopedic Association Scores, but an inferior effectiveness in invalid rate and cure rate.

**Conclusions:**

In the treatment of lumbar disc herniation, Tuina and acupuncture were superior to traction or Chinese herbs, and the effectiveness of traction was the worst. However, considering the limitations of this review, more high-quality trials, especially those comparing Chinese herbs with the other three interventions, should be carried out in the future to further confirm the current findings.

## 1. Introduction

Lumbar disc herniation (LDH) is one of the common spinal disorders, which manifests as low back pain or radiculopathy radiating to the lower limb with a distribution area corresponding to the dermatomes of the related nerve roots [1], influencing the quality of life in patients adversely. With a high prevalence, LDH may affect 1-3% of the general population [2] and becomes an occupational health issue, imposing a heavy burden on social medical security system [3]. Subsequently, its treatments have been paid high attention to in spine department. Both surgical and conservative modalities can be utilized in the treatment of LDH [4, 5]. However, only 15-20% of LDH patients need operative intervention because of severe neurological symptoms [2]. In addition, the effectiveness of surgical procedure relative to conservative treatment remains controversial [2], and some authors suggested that compared with conservative treatment, surgical intervention did not show noteworthy benefit [6]. Thus, conservative treatment is regarded as the first-line choice for LDH.

Traditional Chinese medicine, which evolved over thousands of years, plays an important role in the treatment of LDH, in which Tuina, acupuncture, and Chinese herbs are routinely utilized treatment modalities [7–10]. Tuina, as a form of manipulation treatment under the guidance of the theory of traditional Chinese medicine, involves lots of technical manipulations such as wobbling, pushing, vibrating, and articular moving, performed by a physician using his finger, hand, elbow, knee, or foot [2]. Tuina can exert mechanical effects on skin, muscles, meridians, acupoints, and joints, to relax muscles and tendons, improve circulation, regulate spinal balance, and decrease edema, so it can treat many kinds of disease including LDH [11, 12]. Acupuncture, including manual acupuncture, fire needle, and electroacupuncture, can stimulate the nervous system by regulating meridian and irritating acupoints [13]. Chinese herbs, administered as capsules, tablets, teas, injections, decoctions, or powders [14], can alleviate pain, eliminate inflammation, and reduce spasm [15]. Consequently, the three above-mentioned treatments present with a satisfying effectiveness for LDH [16]. Meanwhile, as one of common physical therapy modalities, lumbar traction plays a therapeutic role by distracting tissues and joints in lumbar spine, which is also widely used in the treatment of LDH [17]. Many authors verified the effectiveness of these four treatments [18]. Some systematic review and meta-analyses [7, 19] have been published to evaluate the effectiveness and safety of these modalities for LDH. However, because of the limitations of the traditional pair-wise meta-analysis, it is difficult to determine which one is the best management in the four modalities.

In recent years, network meta-analysis has been developed. Compared with traditional meta-analysis, it can combine data related to multiple interventions, compare different managements according to indirect information, and generate a ranking for different interventions based on the efficacy [20]. Therefore, we performed a network meta-analysis to evaluate the comparative effectiveness and safety of Tuina, traction, acupuncture, and Chinese herbs, to help physicians better make treatment strategies for patients with LDH.

## 2. Materials and Methods

### 2.1. Data Sources

A medical literature search was performed in the following databases from their inception through May 1st, 2018: PubMed, The Cochrane Library, Embase, Web of Science, the China National Knowledge Infrastructure (CNKI), Chinese Science and Technology Periodical Database (VIP), and Wanfang data. The language of these studies was limited to Chinese and English. The searching was performed using medical subject headings (MeSH) and key words, including “herniated disc”, “herniated disk”, “lumbar disc herniation”, “disc prolapse”, “disk prolapse”, “intervertebral disc displacement”, “intervertebral disk displacement” “slipped disc”, “slipped disk”, “lumbar”, “massage”, “Tuina”, “manipulation”, “traction”, “acupuncture”, “electroacupuncture”, “warm needling”, “Chinese herbs”, “Chinese patent medicine”, “decoction”, “capsule”, “randomization” and “randomized controlled trial”. which were combined in search strategy. Meanwhile, the MeSH and keywords in Chinese including “zhui jian pan tu chu”, “zhui jian pan tuo chu”, “zhui jian pan yi wei”, “zhui jian pan peng chu”, “yao”, “an mo”, “tui na”, “shou fa”, “qian yin”, “zhen ci”, “dian zhen”, “wen zhen”, “zhong yao”, “zhong cheng yao”, “jiao nang”, “tang ji”, “sui ji” and “sui ji dui zhao shi yan” were used for searching literature in Chinese. The details of search strategy are showed in Appendix (page 1-2) (available here). Two investigators performed search independently and imported the identified literature into endnote software to delete the duplications then reviewed the titles and abstracts of the articles to choose the potential ones. The full texts of the chosen articles were checked according to inclusion and exclusion criteria, in which a third investigator checked the divergent articles.

### 2.2. Inclusion Criteria

Trials were included based on the following criteria: (1) randomized controlled trials (RCTs); (2) patients who were diagnosed with LDH based on symptoms, signs, and imaging examination; (3) trials comparing any two of the four interventions including Tuina, traction, acupuncture, and Chinese herbs; (4) trials including at least one comparison in which each of the four interventions was employed as sole management; and (5) trials with complete data.

### 2.3. Exclusion Criteria

The following studies were excluded if (1) literature review; (2) duplicate studies; (3) case reports; (4) animal experiments; (5) studies comparing different types of Tuina, acupuncture, traction or Chinese herbs, such as those comparing manual acupuncture with electroacupuncture, and comparing oblique-pulling manipulation with lumbar rotation manipulation in sitting position.

### 2.4. Data Extraction

Two investigators independently worked for data extraction, and they collected the following information: (1) basic characteristics, including author name, study design, age and gender of patients, intervention, sample size, outcomes, adverse events, and follow-up; (2) primary outcomes, including invalid rate, cure rate, Visual Analogue Score (VAS) and Japanese Orthopedic Association (JOA) Scores.

### 2.5. Quality Assessment

Quality assessment of the included trials was independently performed according to the Cochrane Risk of Bias Tool by two investigators while a third investigator checked disagreements. Risk of bias included the following items: (1) random sequence generation; (2) allocation concealment; (3) blinding of participants and therapist; (4) blinding of outcome assessment; (5) attrition bias; and (6) selective reporting. The judgements on these items were categorized as “low risk of bias”, “high risk of bias”, or “unclear risk of bias”.

### 2.6. Statistical Analysis

A network meta-analysis was carried out using STATA version 14.0. Continuous variables (VAS and JOA) were analyzed using mean difference (MD) and its 95% credible interval (CrI), while dichotomous variables (invalid rate and cure rate) using Odds Ratio (OR). At the beginning of our network meta-analysis, pair-wise meta-analyses were performed, then “mvmeta” package was used to perform the plots of different comparisons, the rank plots based on probabilities and the surface under cumulative ranking (SUCRA) for different endpoints. Furthermore, node-splitting analysis and loop-specific approach were used to evaluate inconsistency, and the Grades of Recommendations Assessment, Development and Evaluation (GRADE) was used to evaluate the importance of the outcomes.

## 3. Result

### 3.1. Identification of Relevant Studies

**Figure 1:**
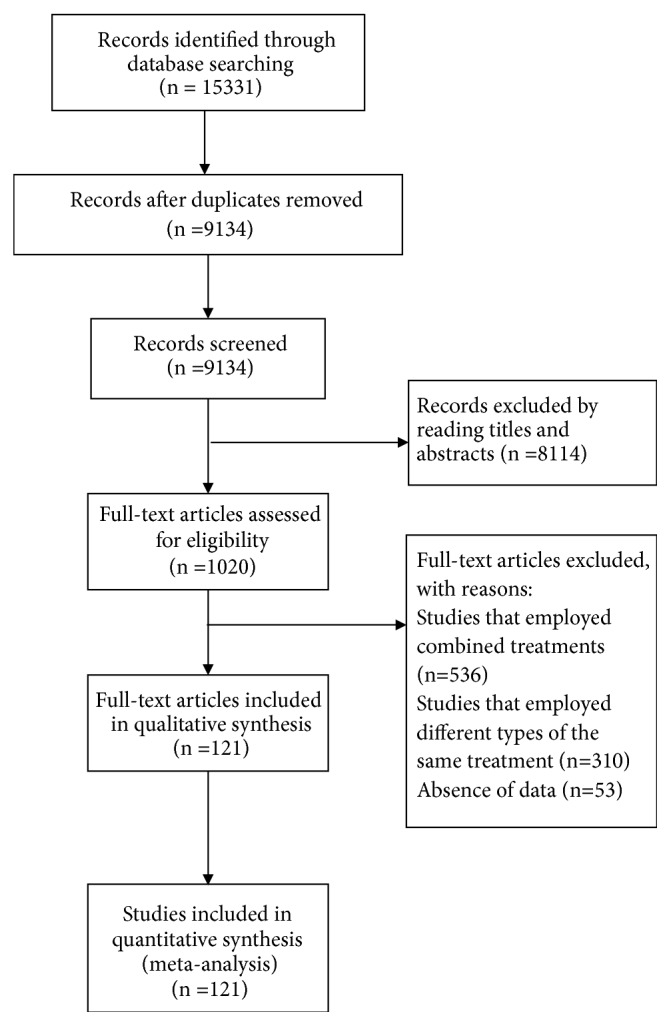
Flow chart of the study selection procedure.

15331 articles were identified in our initial search, from which 6197 articles were excluded for duplications, and 8114 were excluded by reading titles and abstracts. In the remaining 1020 articles, full texts were obtained to check eligibility, in which 536 studied were excluded because of combined treatments in groups, 310 studies were excluded for comparing different types of acupuncture, Tuina, traction, or Chinese herbs in groups, and 53 studies were excluded because of absence of data. Finally, 121 studies were included in our final analysis (Appendix page 2-11).  Figure 1 shows the selection process for relevant studies.

### 3.2. Characteristics of the Included Trials

The review included 121 trials involving 13075 patients, and the sample size ranged from 32 to 900 cases. All the included studies were designed as RCTs. 82 studies were two-arm studies, 35 were three-arm studies, 3 were four-arm studies, and 1 was five-arm study. 31 studies compared Tuina with acupuncture, 34 studies compared Tuina with traction, 29 compared acupuncture with traction, 12 compared Tuina with Chinese herbs, 12 compared acupuncture with Chinese herbs, and 9 compared traction with Chinese herbs. 4241 patients were included in Tuina groups, 3951 patients in acupuncture groups, 3811 patients in traction groups, and 1321 patients in Chinese herbs groups. In terms of the criteria of invalid rate and cure rate, 65 trials employed the criteria of traditional Chinese Medicine syndrome diagnosis, 5 trials used the criteria of clinical research principle of new herbal medicine, 2 trials used the criteria of occupation standard of traditional Chinese medicine, 8 trials used the criteria of Japanese orthopedics association, and the others used the criteria which were similar to above-mentioned standard but did not mention the source. The baseline characteristics of each study are presented in Appendix (page 12-16).

### 3.3. Quality Assessment

The risk of bias assessment is summarized in Appendix (page 17-21). The included patients were randomly assigned to Tuina, Acupuncture, traction or Chinese herbs group. In the generation of randomization sequence, 26 studies used random number tables, 5 studies used network stochastic system and others did not mention the randomization methods. No studies mentioned allocation concealment and method of blinding, and all the studies reported complete data.

### 3.4. The Results of Meta-Analysis

**Figure 2:**
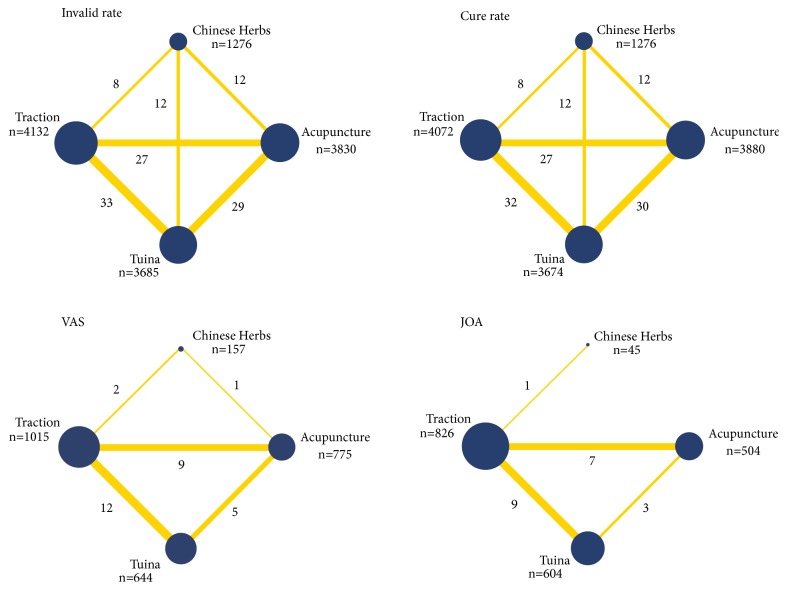
Network of treatment comparisons (Note: width of the lines is proportional to the number of trials comparing every pair of treatments. Size of each circle is proportional to the sample size of interventions. For example, in invalid rate, “8” represents the number of comparisons between traction and Chinese herbs group, “n=4132” represents the sample size of traction group).

**Figure 3:**
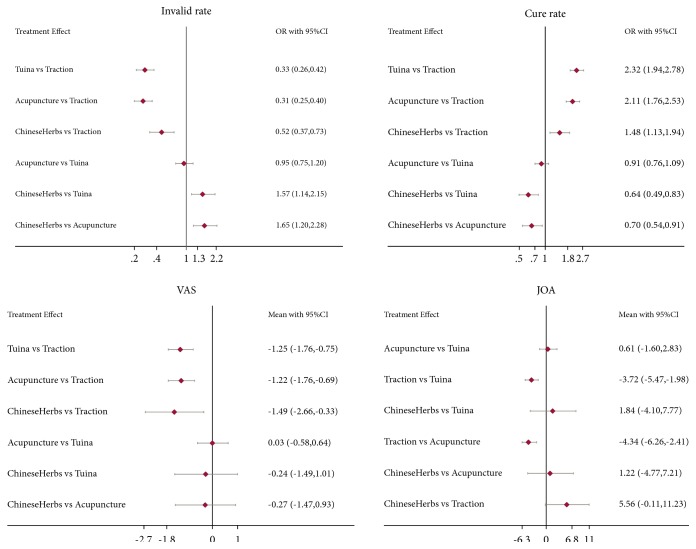
The results of network meta-analysis.

The results of pair-wise meta-analysis are demonstrated in Appendix (page 24).  Figure 2 shows the network of eligible comparisons for invalid rate, cure rate, VAS, and JOA, and Figure 3 shows the results of network meta-analysis.

One hundred and fifteen studies involving 12640 patients and 121 comparisons reported invalid rate (Figure 2). Compared with traction, the interventions including Tuina, acupuncture, and Chinese herbs presented with a significantly lower invalid rate. Meanwhile, the invalid rate was significantly lower in Tuina and acupuncture than Chinese herbs, but no significant difference was found between the two interventions.

In terms of cure rate, 115 studies involving 121 comparisons and 12629 patients were merged for analysis (Figure 2). Compared with traction, the other three treatments showed a significantly higher cure rate. Additionally, the cure rate in Tuina and acupuncture was significantly higher than that in Chinese herbs, but no significant difference was found between the two treatments.

VAS was reported in 29 studies including 2513 patients (Figure 2). When compared with traction, the three other interventions showed a significantly lower VAS score. However, no significant difference was found among the three interventions.

20 studies involving 1979 patients reported JOA scores (Figure 2). As illustrated in Figure 3, no significant difference was found in JOA scores among Tuina, acupuncture, or Chinese herbs. Moreover, JOA scores in Tuina and acupuncture were significantly higher than those in traction, but no significant difference was found between Chinese herbs and traction.

The plots of probability and SUCRA are illustrated in Appendix (page 30-33).  Table 2 shows that Chinese herbs had the highest probability to be the best intervention in VAS and JOA, and Tuina and acupuncture had the highest probability to be the best intervention in cure rate and invalid rate, respectively.

### 3.5. Consistency Analysis

Node-splitting analysis was performed to evaluate the inconsistency by comparing direct and indirect effects, indicating no significant inconsistency (Appendix, page 22-23) and the results were reliable. In addition, the results of Loop-specific approach showed no significant inconsistency in the comparisons of closed circles in outcomes of invalid rate, cure rate or JOA, but significant inconsistency in VAS (Table 1).

### 3.6. GRADE for the Outcome Measurements 

We summarized the GRADE judgements in Appendix (page 25-29). According to the suggestions of GRADE workgroups, we combined the evidences of direct and indirect comparisons and chose a higher level, and the results demonstrated the evidences provided in this review were low or very low.

### 3.7. Adverse Events

13 studies mentioned adverse events, of which 9 studies reported no adverse events, 4 studies reported adverse events, including worsened pain in 22 cases, raised blood pressure in 2 cases, and malposition of facet joints in 12 cases, but no studies mentioned the groups in which the adverse events occurred.

## 4. Discussion

This is the first network meta-analysis to evaluate the efficacy and safety of the four widely utilized treatment modalities for LDH. The results demonstrated that in the four interventions, the effectiveness of lumbar traction was the worst, and the effectiveness of Tuina and acupuncture was better than Chinese herbs in invalid rate and cure rate, but similar as Chinese herbs in VAS and JOA.

The four therapies have different treatment mechanism for LDH. Tuina can decrease the compression of nerve root, relieve the adhesion between nerve root and herniated disc [21], reduce 5-hydroxytryptamine, TNF-*α*, IL-6 [22–24], and improve *β*-EP [25] in patients with LDH. Acupuncture can improve blood circulation and oxygen supply of the nerve roots and sciatic nerve [26], produce rapid analgesic effects [27, 28], and lower plasma adrenocorticotrophic hormone levels [29]. Regarding traction, it can increase disc space height, reduce nucleus pulposus pressure [30], decrease lumbar lordosis and tensile stress on the fibers of annulus fibrosus [31], and promote fluid exchange in disc [32]. In terms of Chinese herbs, it can relieve pain [33] and enhance nerve regeneration, nerve function restoration [34], and neuroprotection [35, 36]. Subsequently, in the treatment of LDH each treatment modality can play an important role in function improvement or pain relief.

However, the four interventions demonstrated different effectiveness, which may be attributed to different mechanism for LDH. The primary symptom of LDH is low back pain and radicular leg pain, so it is critical for patients to relieve pain. In this review, the four outcome measurements including invalid rate, cure rate, VAS, and JOA are used to evaluate pain directly or associated closely with pain. Tuina, acupuncture, and Chinese herbs have direct analgesic effect, but lumbar traction performs its work slowly [37] and does not have direct analgesic effect, so in all outcome measurements the efficacy of lumbar traction was the worst. Additionally, Tuina and acupuncture can exert an immediate analgesic effect [38, 39], so their effectiveness in invalid rate and cure rate was also better than Chinese herbs.

**Figure 4:**
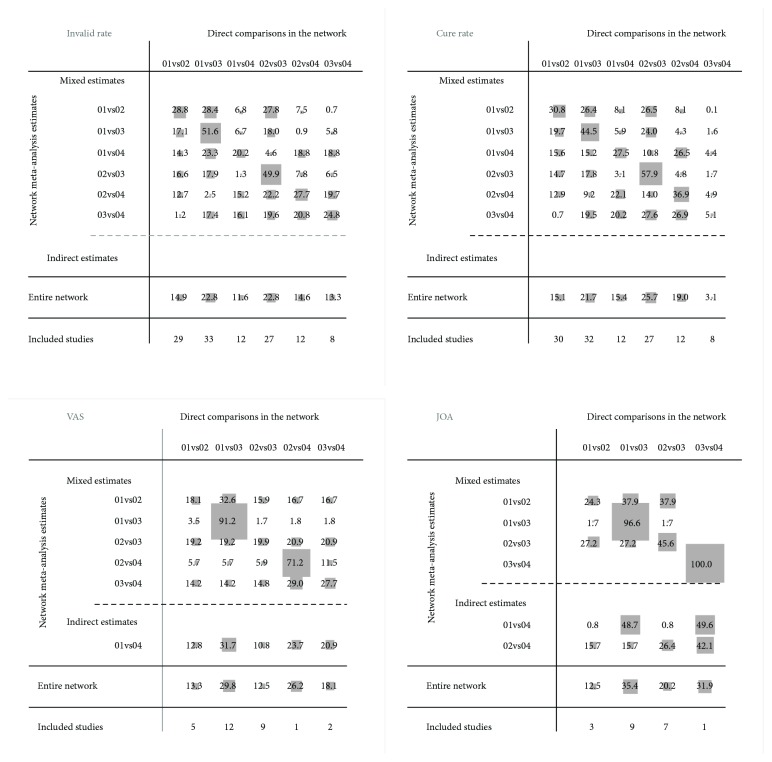
The contribution plots of each outcome (Note: 01=Tuina, 02=Acupuncture, 03=Traction, and 04=Chinese herbs).

**Figure 5:**
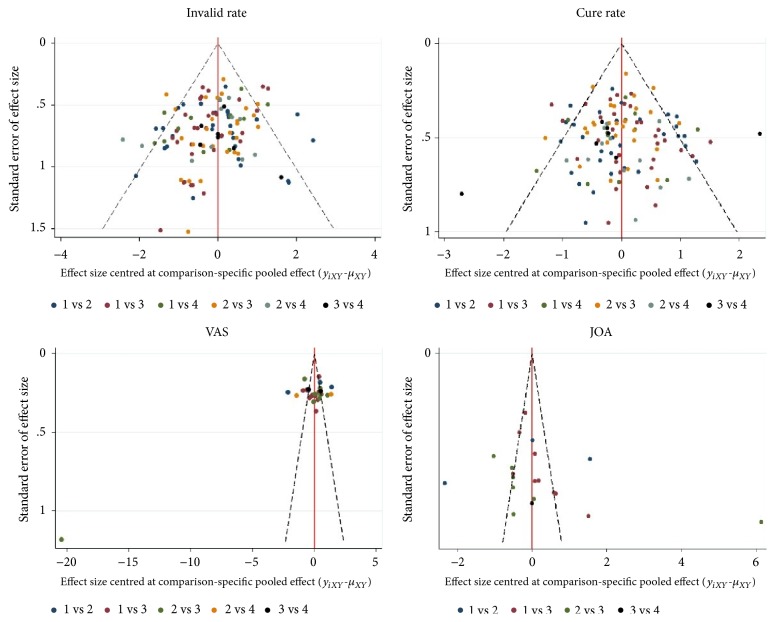
The funnel plots of each outcome (Note: 1=Tuina, 2=Acupuncture, 3=Traction, and 4=Chinese herbs).

In terms of the rank of probability for the four interventions, it is reasonable that Tuina and acupuncture demonstrated a highest probability to be the best intervention in cure rate and invalid rate, but it is noteworthy that Chinese herbs unexpectedly showed superiority in VAS and JOA more than other three interventions. We think the reasons may be attributed to two aspects. First of all, in this review most of trials compared the effectiveness among Tuina, acupuncture, and traction, but the number of comparisons involving Chinese herbs was small. Only four trials compared Chinese herbs with traction or acupuncture in JOA or VAS. As shown in Figure 4, the contribution plot of VAS and JOA showed Chinese herbs accounted for a large proportion, demonstrating the small sample size influenced the total effect and final outcomes adversely. In addition, the funnel plots of VAS and JOA showed the potential report bias (Figure 5). Moreover, the results of Loop-specific approach showed a significant inconsistency existed in VAS, which means the results of indirect comparisons were not consistent with those of direct ones; two comparisons concluded different conclusions in VAS. Subsequently, the findings in favor of Chinese herbs should be interpreted cautiously.

Our review has two methodological strengths. In this research network meta-analysis was carried out to compare the direct and indirect effect of the four treatments, and the SUCRA plot was performed to estimate the ranks of interventions, which may facilitate TCM physicians to make treatment strategies correctly. However, our review has its disadvantages. First, the evidence from GRADE for included outcomes was relatively low. Second, the number and sample size of the trials comparing Chinese herbs with the other three interventions were small. Third, only four outcomes were analyzed in our research, more outcomes such as Euro-Quol questionnaire, health assessment questionnaire (HAQ), and 36-Item Short Form (SF36) were also relevant but not analyzed, because no included studies reported them. Fourth, in some studies the durations of treatment were short, and most of studies did not report the duration of follow-up. These limitations may affect the final outcomes. In addition, most of trials did not mention the adverse events, so the safety of the four treatments could not be evaluated by SUCRA in this review.

## 5. Conclusion

In conclusion, our review suggested, among the four interventions, Tuina and acupuncture were superior to traction or Chinese herbs, and the effectiveness of traction was the worst in the treatment of LDH. However, considering the limitations of this study, more high-quality trials, especially those comparing Chinese herbs with the other three interventions, should be carried out in the future to further confirm the current conclusions.

## Figures and Tables

**Table 1:** Loop-specific approach.

Outcomes	Loop	ROR	Z_value	P_value	95% CI	Loop_Heterog_tau2
Invalid rate	Tui-Tra-Chi	1.844	1.518	0.129	(1.00, 4.06)	0.207
	Tui-Acu-Chi	1.524	0.929	0.353	(1.00, 3.71)	0.490
	Tui-Acu-Tra	1.406	1.284	0.199	(1.00, 2.37)	0.240
	Acu-Tra-Chi	1.242	0.585	0.558	(1.00, 2.57)	0.096
Cure rate	Tui-Tra-Chi	1.492	1.158	0.247	(1.00, 2.94)	0.155
	Tui-Acu-Chi	1.425	1.893	0.058	(1.00, 2.06)	0.130
	Tui-Acu-Tra	1.223	0.652	0.515	(1.00, 2.24)	0.185
	Acu-Tra-Chi	1.161	0.408	0.683	(1.00, 2.38)	0.259
VAS	Tui-Acu-Tra	10.817	1.233	0.218	(1.00, 476.46)	1.305
	Acu-Tra-Chi	23.315	0.322	0.748	(1.00, 277137.09)	2.510
JOA	Tui-Acu-Tra	1.629	0.368	0.713	(1.00, 21.93)	1.715

Note: loop-specific approach is used to check the inconsistency which aims at the closed loop. In this analysis, ROR is close to 1, indicating no significant difference between direct and indirect effects. VAS=visual analogue score, JOA=Japanese Orthopedic Association Score, Tui=Tuina, Tra=Traction, Acu=Acupuncture, Chi=Chinese herbs.

**Table 2:** The results of SUCRA and probability.

Treatments/outcomes	SUCRA	PrBest	MeanRank
Invalid rate			

Tuina	77.3	32.1	1.7
Acupuncture	89.3	67.8	1.3
Traction	0.0	0.0	4.0
Chinese herbs	33.5	0.1	3.0

Cure rate			

Tuina	95.1	85.2	1.1
Acupuncture	71.4	14.7	1.9
Traction	0.0	0.0	3.0
Chinese herbs	33.6	0.1	4.0

VAS			

Tuina	63.7	25.3	2.1
Acupuncture	59.8	16.6	2.2
Traction	0.3	0.0	4.0
Chinese herbs	76.3	58.1	1.7

JOA			

Tuina	52.0	10.6	2.4
Acupuncture	68.8	26.2	1.9
Traction	1.1	0.0	4.0
Chinese herbs	78.2	63.2	1.7

Notes: data are probability in the rows of “SUCRA” and “PrBest”. SUCRA= surface under the cumulative ranking, PrBest= the best probability, VAS=Visual analogue score, JOA=Japanese Orthopedic Association Score.

## Supplementary Materials

Supplementary Materials The supplementary appendix has 33 pages, including search strategy, references list of included studies, characteristics of included studies, risk of bias of included studies, node-splitting analysis, pair-wise meta-analysis, GRADE for outcome measurements, SUCRA, and probability plots.
