# Supplementary material for: Comparisons of the Effectiveness and Safety of Tuina, Acupuncture, Traction, and Chinese Herbs for Lumbar Disc Herniation: A Systematic Review and Network Meta-Analysis
Source: Evid Based Complement Alternat Med. 2019 Mar 20;2019:6821310. doi: 10.1155/2019/6821310 (PMC6446119; doi:10.1155/2019/6821310)

**Supplementary Appendix**

**Contents**

[1 Search strategy 1](#_Toc519669160)

[2 References list of included studies 2](#_Toc519669161)

[3 Characteristics of included studies 12](#_Toc519669162)

[4 Risk of bias of included studies 17](#_Toc519669163)

[5 Node-splitting 22](#_Toc519669164)

[6 Results of pair-wise meta-analyses 24](#_Toc519669165)

[7 GRADE for outcome measurements 25](#_Toc519669166)

[8 SUCRA and probability plots 31](#_Toc519669167)

## 1 Search strategy

#1 Tuina (Tui na)

#2 Massage (An mo)

#3 Manipulation (Shou fa)

#4 Traction (Qian yin)

#5 Acupuncture (Zhen ci)

#6 electroacupuncture (Dian zhen)

#7 warm needling (Wen zhen)

#8 Chinese herbs (Zhong yao)

#9 Chinese patent medicine (Zhong cheng yao)

#10 capsule (Jiao nang)

#11 decoction (Tang ji)

#12 herniated disc

#13 herniated disk

#14 lumbar disc herniation (Zhui jian pan tu chu)

#15 slipped disc

#16 slipped disk (Zhui jian pan peng chu)

#17 disc prolapse

#18 disk prolapse (Zhui jian pan tuo chu)

#19 intervertebral disc displacement

#20 intervertebral disk displacement (Zhui jian pan yi wei)

#21 lumbar (yao)

#22 randomization (sui ji)

#23 randomized controlled trial (sui ji dui zhao shi yan)

#24 (#1 or #2 or #3) and #4 and (#12 or #13 or #14 or #15 or #16 or #17 or #18 or #19 or #20) and #21 and (#22 or #23)

#25 (#1 or #2 or #3) and (#5 or #6 or #7) and (#12 or #13 or #14 or #15 or #16 or #17 or #18 or #19 or #20) and #21 and (#22 or #23)

#26 (#1 or #2 or #3) and (#8 or #9 or #10 or #11) and (#12 or #13 or #14 or #15 or #16 or #17 or #18 or #19 or #20) and #21 and (#22 or #23)

#27 #4 and (#5 or #6 or #7) and (#12 or #13 or #14 or #15 or #16 or #17 or #18 or #19 or #20) and #21 and (#22 or #23)

#28 #4 and (#8 or #9 or #10 or #11) and (#12 or #13 or #14 or #15 or #16 or #17 or #18 or #19 or #20) and #21 and (#22 or #23)

#29 (#5 or #6 or #7) and (#8 or #9 or #10 or #11) and (#12 or #13 or #14 or #15 or #16 or #17 or #18 or #19 or #20) and #21 and (#22 or #23)

## 2 References list of included studies

1. Ao 2008

J.B. Ao, J. Chen, J.P. Mu, L. Peng, J.M. Cheng, L.Z. Zhou, J. Wang. Clinical observation of 40 cases with lumbar disc herniation treated with electroacupuncture plus three-dimensional traction. Journal of Emergency in Traditional Chinese Medicine 17 (2008), pp. 626-627+629.

2. Cai 2012

A.S. Cai. Forty-three cases with lumbar disc herniation treated with acupuncture. Shanxi Journal of Traditional Chinese Medicine 33 (2012), pp.1397.

3. Cao 2016

Z.Q. Cao. Effectiveness of Chinese medicine, acupuncture and Tuina for lumbar disc herniation. World Latest Medicine Information 16 (2016), pp. 149, 151.

4. Chen 2000

R.H. Chen, S.R. Chen. The effectiveness of acupuncture combined with Tuina in treating lumbar disc herniation. Modern Rehabilitation 4 (2000), pp. 766.

5. Chen 2012

F.H. Chen, P. Zhao. Clinical observation of 36 cases with lumbar disc herniation treated using Ji tong ling. Zhejiang Journal of Traditional Chinese Medicine 47 (2012), pp. 741.

6. Chen 2014

G.X. Chen. The discussion and experience in the treatment lumbar disc herniation. For all Health 8 (2014), pp. 53-54.

7. Dai 2015

D.X. Dai. Effectiveness of acupuncture for lumbar disc herniation. Chinese Journal of Clinical Rational Drug Use 8 (2015), pp. 15-16.

8. Ding 2014

Y. Ding, H.N. Liu, S.L. He. The effectiveness of Tuina in treating lumbar disc herniation. Chinese Journal of Clinical Rational Drug Use 7 (2014), pp. 123-124.

9. Dong 2010

T. Dong. The clinical study of massage in treating lumbar disc herniation. Modern Medicine & Health 26 (2010), pp. 1393-1395.

10. Duo 2016

X. Duo, X.X. Ba. Clinical observation on the treatment of lumbar intervertebral disc herniation with warm acupuncture and moxibustion. Journal of New Chinese Medicine 48 (2016), pp. 99-101.

11. Fan 2009

Y. Fan, L.F. Xue, X.F. Meng. The efficacy of warm acupuncture and moxibustion in treating lumbar disc herniation. Modern Distance Education of Chinese Medicine 7 (2009), pp. 114-115.

12. Fang 2014

X.M. Fang. Clinical observation of Tuina for lumbar disc herniation. Nei Mongol Journal of Traditional Chinese Medicine 4 (2014), pp. 73-74.

13. Feng 2008

H.Y. Feng, S.L. Deng. The observation on effect of treatment for lumbar disc herniation with modified aconite decoction. The Journal of Traditional Chinese Orthopedics and Traumatology 20 (2008), pp. 66.

14. Fu 2011

X.S. Fu. Comparative study on the efficacy of 240 patients with lumbar disc herniation treated by acupuncture and Chinese massage. World Journal of Integrated Traditional Chinese medicine and Western Medicine 6 (2011), pp. 1058-1060.

15. Gao 2013

J.C. Gao, A.X. Liu. A randomized controlled trial of warming needle moxibustion in the treatment of lumbar disc herniation. Journal of Practical Traditional Chinese Internal Medicine 27 (2013), pp. 67-68.

16. Geng 2008

P. Geng. Observation on the effectiveness of warm acupuncture in the treatment of lumbar disc herniation. Journal of Clinical Acupuncture and Moxibustion 24 (2008), pp. 29-30.

17. Gong 2001

R.L. Gong. Observation of massage in treating lumbar disc herniation. Chinese Manipulation & Qi Gong therapy 17 (2001), pp. 9-10.

18. Gu 2011

F. Gu, Y. Huang, M. Fang. The effectiveness of Tuina on soft tissue tension in patients with lumbar disc herniation. Academic Journal of Shanghai University of Traditional Chinese Medicine 25 (2011), pp. 44-46.

19. Gu 2013

F. Gu, Y. Huang, J.L. Wang, M. Wei, L. Zheng. Effect of three-step massage therapy on lumbar curvature. China Medical Herald 10 (2013), pp. 115-117+120.

20. Guo 2014

R.R. Guo. The comparison between acupuncture and Tuina in treating lumbar disc herniation. Thesis of Master Degree, Beijing university of Chinese Medicine, Beijing, China, 2014.

21. Han 2009

L. Han. The mechanical visualization training and clinical study of Chinese manipulation in treating lumbar disc herniation. Thesis of Master Degree, China Academy of Chinese Medical Sciences, Beijing, China, 2009.

22. Hao 2017

H. Hao. Clinical study on the patients with lumbar intervertebral disc protrusion treated by acupuncture and moxibustion. Systems Medicine 2 (2017), pp. 135-136+139.

23. He 2011

W. He, S.T. Fang, S.L. Wang, G.X. Yang. Clinical trial in the treatment of lumbar disk herniation by method of straight leg raise. Chinese Journal of Traditional Medical Traumatology & Orthopedics 19 (2011), pp. 34-35.

24. He 2013

X. He. Clinical experience for treating lumbar disc herniation by acupuncture. Clinical Journal of Chinese Medicine 5 (2013), pp. 34-35.

25. Hu 2014

J.Q. Hu. Analysis of the treatment of pain resulting from lumbar disc herniation using acupuncture. Nei Mongol Journal of Traditional Chinese Medicine 27 (2014), pp. 68-69.

26. Hu 2015

J.S. Hu, X.B. Xi, S.Y. Wan, R.T. Shi, F.Y. Li. Clinical observation on the treatment of lumbar disc herniation with Wei’s manipulation and Chinese fumigation. Chinese Journal of Traditional Medical Traumatology & Orthopedics 23 (2015), pp. 8-11.

27. Huang 2008

B.G. Huang, H. Huang. Comparison between Chinese manipulation and traction in treating lumbar disc herniation. Medical Information 21 (2008), pp. 925-926.

28. Huang 2013^a^

P.T. Huang, L. Wang, Z.L. Wang, J. Su, Y.F. Liu, P. Zhang, C. Ge. Methods of 3 small fixed- point manipulation in treating lumbar intervertebral disc protrusion. Zhejiang Journal of Traditional Chinese Medicine 37 (2013), pp. 1027-1029.

29. Huang 2013^b^

Z.D. Huang, Y.Y. Hu, B. Liang. Observation of acupuncture combined with chiropractic in treating lumbar intervertebral disc herniation of blood stasis type. Sichuan journal of Traditional Chinese Medicine 31 (2013), pp. 139-141.

30. Huang 2015

J.W. Huang, G.S. Zhang, P.S. Tan, H. Zhong, A.L. Guo, H.L. Wang, X.R. Chang. Clinical observation of 40 patients with lumbar disc herniation treated using oblique-pulling manipulation. Hunan Journal of Traditional Chinese Medicine 31 (2015), pp. 62-64.

31. Jia 2015

S.T. Jia. Clinical studies of lumbar disc herniation treated with acupuncture and Chinese medicine. Thesis of Master Degree, Hebei Medical University, Hebei, China, 2015.

32.Jiang 2005

B. Jiang, Clinical observation of patients with lumbar disc herniation treated by jing yao tong I decoction. Thesis of Master Degree, Liaoning college of Traditional Chinese Medicine, Liaoning, China, 2015.

33. Jiang 2011

H. Jiang. Observation of 74 patients with lumbar disc herniation treated with Chinese manipulation. Shandong Medical Journal 51 (2011), pp. 102-103.

34. Jiang 2014

H.G. Jiang. Analysis of the effectiveness and safety of acupuncture on lumbar intervertebral disc herniation. Shenzhen Journal of Integrated Traditional Chinese and Western Medicine 24 (2014), pp. 29-30.

35. Jiao 2005

B.L. Jiao, Q.L. Dou, F. Yang. Observation of 30 patients with lumbar disc herniation treated using Du Zhong decoction. Shanxi Journal of Traditional Chinese Medicine 26 (2005), pp. 1055-1056.

36. Ju 2014

Y.D. Ju, L.G. Qin. Clinical observation of 146 patients with lumbar disc herniation treated using Chinese manipulation. China & Foreign Medicine 26 (2014), pp. 18-19.

37. Lei 2018

H.P. Lei, J.H. Chen, W.G. Zhang, S.B. Yang. Clinical observation on electroacupuncture combined with massage in the treatment of lumbar disc herniation. Hubei University Journal of Chinese Medicine 20 (2018), pp. 96-99.

38. Li 2003

J. Li. A study of effectiveness and mechanism of Tuina on lumbar disc herniation. Thesis of Master Degree, Shandong University of Traditional Chinese Medicine, Shandong, China, 2003.

39. Li 2010

W.L. Li, J.Y. Huang. Observation of 100 patients with lumbar disc herniation treated using Chinese manipulation. Modern distance education of Chinese medicine 8 (2010), pp. 39-40.

40. Li 2012

X.M. Li, Y.D. Liu. Clinical observation of Yao tong an I treating lumbar disc herniation. Clinical Journal of Chinese Medicine 4 (2012), pp. 23-25.

41. Li 2013

Y.J. Li. Clinical observation of Chinese manipulation treating lumbar disc herniation. Guide of Chinese Medicine 11 (2013), pp. 283-284.

42. Li 2014

M.W. Li. Analysis of the effect of acupuncture in the treatment of lumbar intervertebral disc herniation of foot drop type. Journal of Chinese Medicine 29 (2014), pp. 1539-1540.

43. Li 2016

J.C. Li. Observation of 80 patients with lumbar disc herniation treated with Chinese manipulation. Modern Health Care 14 (2016), pp. 177.

44. Liang 2015

Z.L. Liang. Treating lumbar intervertebral disc herniation using Yao san zhen and SNAGS techniques. Thesis of Master Degree, Guangzhou University of Chinese Medicine, Guangdong, China, 2015.

45. Liao 2010

L.L. Liao, R.Z. Jiang, D.T. Huang. Observation of the efficacy of Chinese medicine combined with acupuncture in treating lumbar disc herniation. Sichuan journal of Traditional Chinese Medicine 28 (2010), pp. 111-112.

46. Lin 2013

J. Lin. The influence of electroacupuncture on the level of IL-6 and β-EP in patients with lumbar disc herniation. China Practical Medicine 8 (2013), pp. 125-127.

47. Liu 2008

Z.G. Liu. Clinical study of three-step manipulation in treating lumbar disc herniation. Thesis of Master Degree, Hubei College of Traditional Chinese Medicine, Hubei, China, 2008.

48. Liu 2009

L. Liu, L.G. Liu, M. Lv, W.J. Ran. Observation on the effectiveness of electroacupuncture combined with Chinese herbal medicine in the treatment of lumbar intervertebral disc prolapse of Yang deficiency and cold coagulation type. Chinese Acupuncture & Moxibustion 29 (2009), pp. 626-628.

49. Liu 2012

H.Y. Liu. The efficacy of acupuncture in treating lumbar disc herniation. World Health Digest Medical Periodical 9 (2012), pp. 203.

50. Liu 2013^a^

J.S. Liu. Clinical study on the comprehensive therapy of Chinese medicine treating lumbar disc herniation. Clinical Journal of Chinese Medicine 5 (2013), pp. 56-57.

51. Liu 2013^b^

Z.Q. Liu. Observation of 67 patients with lumbar disc herniation treated with acupuncture. Guide of China Medicine 11 (2013), pp. 264.

52. Liu 2014

Y.F. Liu. Clinical observation on 28 cases of lumbar disc herniation treated by electroacupuncture. Yunan Journal of Traditional Chinese Medicine and Materia Medica 35 (2014), pp. 58.

53. Liu 2016

Q.M. Liu. Clinical observation of the effectiveness of Huo Luo Xiao Ling Dan on patients with lumbar disc herniation. World latest medicine information 16 (2016), pp. 136.

54. Lun 2006

S.F. Lun, L.M. Lei, J. Pang. Clinical observation of 75 cases of lumbar disc herniation treated by Tuina. Journal of Guangxi University of Traditional Chinese Medicine 9 (2006), pp. 51-53.

55. Luo 2008

P.A. Luo, X.C. Yan. Clinical observation of patients with lumbar disc herniation treated with warm acupuncture. China medical herald 5 (2008), pp. 92-93.

56. Luo 2009

J.F. Luo, B.L. Luo. Clinical observation on 40 cases of lumbar intervertebral disc prolapse treated by Du Huo Ji Sheng decoction. Guiding Journal of Traditional Chinese Medicine and Pharmacy 15 (2009), pp. 35-36.

57. Ma 2016

Z.J. Ma, Y.J. Chen, Y.R. Liu, Y.J. Zhao, L.X. Ma, P.C. Ji. Clinical observation of warm acupuncture and moxibustion for treating lumbar disc herniation of blood stasis type. Hebei Medicine 22 (2016), pp. 1552-1554.

58. Mao 2009

S.G. Mao, Y.X. Guo, Q.A. Zhao, X.J. Kong. Clinical observation of 160 cases of lumbar disc herniation treated using Chinese manipulation. Chongqing Medicine 38 (2009), pp. 2120-2121.

59. Meng 2010

D.Y. Meng. Comparison between Chinese medicine and Tuina for lumbar disc herniation. China practical medicine 5 (2010), pp. 143-144.

60. Meng 2013

J. Meng. Comparison of efficacy between tuina and Chinese medicine in treating lumbar disc herniation. Health required 12 (2013), pp. 92-93.

61. Ning 2015

G.L. Ning. Efficacy of oblique-pulling manipulation on lumbar function in patient with lumbar disc herniation. Thesis of Master Degree, Henan College of Traditional Chinese Medicine, Henan, China, 2015.

62. Quan 2006

X.B. Quan, G.Q. Lu, D.C. Cao. X-ray observation of flexion and extension function in patients with lumbar disc herniation treated using Chinese manipulation. Zhejiang Journal of Integrated Traditional Chinese and Western Medicine 16 (2006), pp. 390-391.

63. Sha 2011

L.Q. Sha. Clinical study of modified lumbar rotation manipulations. Guide of China Medicine 9 (2011), pp. 141-142.

64. Sha 2017

H.B. Sha. The effect of Tuina on lumbar disc herniation. Contemporary Medical Symposium 15 (2017), pp. 33-34.

65. She 2016

T.T. She, H.Y. Zhang, Z.J. Wu, Y.M. Tu, Y.G. Guo, K.M. Hu. Clinical effectiveness of electroacupuncture for lumbar disc herniation. Sichuan journal of Traditional Chinese Medicine 34 (2016), pp. 179-180.

66. Shen 2016

D.L. Shen and W.L. Wang. Treating 135 patients with lumbar disc herniation by three-step, three-position, and nine-method manipulation. Western Journal of Traditional Chinese Medicine 29 (2016), pp. 121-123.

67. Shi 2010

S.Y. Shi. The clinical study on lumbar disc herniation of stasis blood type treated by two-step and ten- method manipulations. Thesis of Master Degree, Changchun University of Traditional Chinese Medicine, Jilin, China, 2010.

68. Song 2005

T.B. Song. Standard and quantitative study of oblique pulling manipulation for lumbar disc herniation. Thesis of Master Degree, Chinese Academy of Traditional Chinese Medicine, Beijing, China, 2005.

69. Song 2010

Z.X. Song. Clinical study on lumbar disc herniation treated by three-step treatment. Thesis of Master Degree, Changchun University of Traditional Chinese Medicine, Jilin, China, 2010.

70. Su 2013

D.J. Su. Clinical study on the efficacy of electroacupuncture combined with massage for lumbar disc herniation. Thesis of Master Degree, Hubei University of Chinese Medicine, Hubei, China, 2013.

71. Sun 2012

H. Sun. Recent analgesic effect of warm acupuncture on patients with lumbar disc herniation. Thesis of Master Degree, Chengdu University of Traditional Chinese Medicine, Sichuan, China, 2012.

72. Sun 2014^a^

H.C. Sun. Observation of patients with lumbar disc herniation treated by warm acupuncture. Sichuan journal of Traditional Chinese Medicine 32 (2014), pp. 150-151.

73. Sun 2014^b^

J. Sun. Clinical Observation on electroacupuncture combined with traction and manipulation for lumbar disc herniation. Chinese Manipulation & Rehabilitation Medicine 5 (2014), pp. 12-13.

74. Sun 2016

J.B. Sun. Observation of lumbar disc herniation treated by Caiqiao manipulations. Journal of Practical Traditional Chinese Medicine 32 (2016), pp. 817-818.

75. Tang 2009

S.D. Tang, G.H. Chen, Z.M. Lv. Efficacy of abdominal acupuncture on lumbar disc herniation. World Journal of Integrated Traditional and Western Medicine 4 (2009), pp. 572-573.

76. Tang 2012

X.Z. Tang, H.T. Ding, F. Yang, J. Chen. Clinical effect of three-position, eight-step tuina in treating lumbar disc herniation. China Journal of Traditional Chinese Medicine and Pharmacy 27 (2012), pp. 1464-1466.

77. Tu 2014

X.S. Tu. Observation of 60 patients with lumbar disc herniation treated by tuina. Yunnan Journal of Traditional Chinese Medicine and Materia Medica 35 (2014), pp. 87-88.

78. Wang 2007

Y.L. Wang, W.Y. Zhang. Observation of 110 patients with lumbar disc herniation treated by combined therapies. Journal of Changchun University of Traditional Chinese Medicine 23 (2007), pp. 75-76.

79. Wang 2010

L.H. Wang. Clinical study on patients with lumbar intervertebral disc protrusion treated with oblique-pulling manipulation. Thesis of Master Degree, Hunan University of Traditional Chinese Medicine, Hunan, China, 2010.

80. Wang 2013

L.X. Wang, Q. Wang. Clinical study of 130 cases of lumbar disc herniation treated with massage. Guide of China Medicine 11 (2013), pp. 282-283.

81. Wang 2016

C.E. Wang. Clinical observation of patients with lumbar intervertebral disc herniation treated by massage. World Latest Medicine Information 16 (2016), pp. 141.

82. Wang 2017

X.H. Wang. Clinical research on Tong du Spinal manipulation in the treatment of lumbar disc herniation. Thesis of Master Degree, Shanxi Province Institute of Traditional Chinese Medicine, Shanxi, China, 2017.

83. Wen 2010

D.D. Wen. Clinical observation of lumbar disc herniation treated with four-step manipulation. Guiding Journal of Traditional Chinese Medicine and Pharmacy 16 (2010), pp. 55-56.

84. Wu 2007

J.C. Wu. Clinical observation of 267 cases of lumbar disc herniation treated with traction and manipulation. Jilin Journal of Traditional Chinese Medicine 27 (2007), pp. 33-34.

85. Wu 2011

W.F. Wu, X.L. Chen, M.L. Wu, X.M. Zhong, R.H. Luo, M.J. Xu, B Nie, K. Xu, M.J. Lin. Effect of acupuncture and massage on immune system of patients with lumbar disc herniation. Chinese Journal of Gerontology 31 (2011), pp. 1135-1137.

86. Wu 2015^a^

A.J. Wu. Efficacy of warming needle and moxibustion in treating lumbar disc herniation. Clinical Journal of Chinese Medicine 7 (2015), pp. 25-26.

87. Wu 2015^b^

X.P. Wu. Clinical effect of acupuncture in treating lumbar disc herniation. The Medical Forum 19 (2015), pp. 4091-4092.

88. Xie 2009

S.S. Xie. Comparison between manipulation and traction in treating lumbar disc herniation. Guiding Journal of Traditional Chinese Medicine and Pharmacy 15 (2009), pp. 60-61.

89. Xie 2011

D.F. Xie. Comparison between electroacupuncture and traction in treating lumbar disc herniation. Guide of China Medicine 9 (2011), pp. 305-306.

90. Xiong 2013

J. Xiong, J.B. Fang, X. Chen, L.Y. Jiang, D.Y. Zhou. Clinical effectiveness of acupuncture, massage, acupuncture combined with massage for lumbar disc herniation. Guide of China Medicine 11 (2013), pp. 300-301.

91. Xiong 2016

X.Q. Xiong. The efficacy of acupuncture for lumbar disc herniation. Contemporary Medical Symposium 14 (2016), pp. 11-12.

92. Xuan 2013

J.Y. Xuan Comparison between traction and Chinese medicine in treating lumbar disc herniation. The Medical Journal of Industrial Enterprise 26 (2013), pp. 360-361.

93. Xue 2017

H.X. Xue. Clinical efficacy of double-doctor positioned oblique-pulling manipulation in the treatment of lumbar disc herniation. Acta Chinese Medicine 32 (2017), pp. 477-479.

94. Yang 2004

J.G. Yang. The combination of Tuina and Chinese medicine in treating lumbar disc herniation. Chinese Journal of Rehabilitation 19 (2004), pp. 354.

95. Yang 2006

L. Yang, C.L. Cui. The observation of 60 cases of lumbar disc herniation treated with electroacupuncture. The Journal of Cervicodynia and Lumbodynia 27 (2006), pp. 144.

96. Yang 2010

X.F. Yang. Clinical study of gravity massage on lumbar disc herniation. Thesis of Master Degree, Changchun University of Traditional Chinese Medicine, Jilin, China, 2010.

97. Yang 2014

J.X. Yang. Observation on lumbar disc herniation treated by acupuncture. Asia-Pacific Traditional Medicine 10 (2014), pp. 70-71.

98. Yang 2016^a^

J.W. Yang. Clinical effect of warm acupuncture in the treatment of lumbar disc herniation. Medical Equipment 29 (2016), pp. 80-81.

99. Yang 2016^b^

X.S. Yang. Clinical research of acupuncture and massage in the treatment of lumbar disc herniation. Shenzhen Journal of Integrated Traditional Chinese and Western Medicine 26 (2016), pp. 48-50.

100. Ye 2012

J.X. Ye. Therapeutic effect of acupuncture, traction and massage on lumbar disc herniation. Nutrition and Health Care in China 4 (2012), pp. 132.

101. Yu 2008^a^

L. Yu, Y.B. Huang, H.L. Chen. Sixty-two patients with lumbar disc herniation treated with three-dimensional Tuina. Jiangxi Journal of Traditional Chinese Medicine 5 (2008), pp. 36.

102. Yu 2008^b^

L. Yu, H.L. Chen, Y.M. Li Clinical observation of fixed-point reduction manipulation in treating lumbar disc herniation. Chinese Manipulation & Qi Gong therapy 24 (2008), pp. 17-19.

103. Yun 2014

B.S. Yun, Z.H. Kong, T. Fu, H.Y. Zhai, Y.Z. Liu, G.B. Ma, S.H. Guo, J.G. Lian. Innovative application of cocktail therapy of traditional Chinese medicine on lumbar intervertebral disc herniation. Chinese Community Doctors 30 (2014), pp. 106-107.

104. Zhan 2017

Z.J. Zhan Clinical study on patients with lumbar intervertebral disc herniation treated by He’s external application of traditional Chinese medicine. Thesis of Master Degree, Guangzhou University of Chinese Traditional Medicine, Guangdong, China, 2017.

105. Zhang 2002

D.W. Zhang, X.F. Liang, Q.J. Wang. Clinical comparison between acupuncture and massage for the treatment of lumbar intervertebral disc protrusion. Shanghai Journal of Acupuncture and Moxibustion 21 (2002), pp. 22-23.

106. Zhang 2010

J. Zhang, L. Han, F. Wang, D. Yu, M. Lu, D.K. Lin, T.B. Song, J.H. Lin, S.C. Sun. Clinical effect of oblique-pulling manipulation in the treatment of lumbar intervertebral disc herniation. China Journal of Orthopedics & Traumatology 23 (2010), pp. 84-86.

107. Zhang 2012

L.J. Zhang. Observation of lumbar disc herniation treated by Chinese medicine. Guide of China Medicine 10 (2012), pp. 221-222.

108. Zhang 2014

Y.X. Zhang. Clinical observation of lumbar disc herniation treated by acupuncture and moxibustion. Chinese Journal of Trauma and Disability Medicine 22 (2014), pp. 43.

109. Zhang 2015

J. Zhang, X.P. Li. Clinical effect of acupuncture combined with massage in the treatment of lumbar disc herniation. China Health Standard Management 6 (2015), pp. 123-124.

110. Zhang 2016^a^

J.A. Zhang. Clinical observation of acupuncture and moxibustion therapy in the treatment of lumbar disc herniation. Journal of Hubei University of Chinese Medicine 18 (2016), pp. 87-89.

111. Zhang 2016^b^

S.H. Zhang. Clinical effect of Chinese manipulation on lumbar disc herniation. For all health 11 (2016), pp. 25-26.

112. Zhang 2016^c^

Y. Zhang. Clinical observation of acupuncture combined with medicine for lumbar disc herniation of cold-dampness type. Thesis of Master Degree, Guangzhou University of Traditional Chinese Medicine, Guangdong, China, 2016.

113. Zhang 2017

L. Zhang, Q. Gao, G. Wang, Y.L. Tian, Y.L. Liang, M.L. Sun. Comparison of the short-term efficacy between inner heating dry needle and lumbar traction in treating lumbar disc herniation. Chinese Journal of Health Care Medicine 19 (2017), pp. 122-124.

114. Zhao 2007^a^

C.W. Zhao. Clinical study of lumbar disc herniation treated by traction and pulling manipulation. Thesis of Master Degree, Changchun University of Traditional Chinese Medicine, Jilin, China, 2007.

115. Zhao 2007^b^.

Q. Zhao. Clinical study of lumbar disc herniation treated by prone pulling manipulation. China Journal of Traditional Chinese Medicine and Pharmacy 22 (2007), pp. 15-17.

116. Zhao 2008

J.Y. Zhao. Effectiveness of electroacupuncture in treating lumbar disc herniation. Journal of Clinical Acupuncture and Moxibustion 24 (2008), pp. 10-11.

117. Zhao 2011

F.L. Zhao. Observation of lumbar disc herniation treated by comprehensive treatments. Shanxi Journal of Traditional Chinese Medicine 27 (2011), pp. 32-33.

118. Zhi 2001

L.X. Zhi. Clinical study of lumbar intervertebral disc herniation treated by massage, acupuncture and traction. Hebei Medicine 7 (2001), pp. 1069-1071.

119. Zhou 2001

X.D. Zhou. Observation of 308 cases of lumbar disc herniation treated by traction and Tuina. Journal of Medical College of Jing gang shan 8 (2001), pp. 95-96.

120. Zhou 2010

M.H. Zhou, B.L. Zhang. Observation of 51 cases of lumbar disc herniation treated by acupuncture and Chinese medicine. Zhejiang Journal of Traditional Chinese Medicine 45 (2010), pp. 210-211.

121. Zhu 2012

Z.F. Zhu. Clinical analysis of lumbar intervertebral disc herniation treated with Chinese medicine and acupuncture. Clinical Journal of Chinese Medicine 4 (2012), pp. 87-88.

## 3 Characteristics of included studies

| Study | Included arms (n) | Total arms(n) | Gender  (m/f) | Mean age (y) |  | Duration of disease | Sample size | Interventions | Treatment duration | Outcomes |
| --- | --- | --- | --- | --- | --- | --- | --- | --- | --- | --- |
| Ao 2008 | 2 | 3 | N/A | 39.7 |  | 5.5±2.3 m | 40 vs 40 | (2) vs (3) | 28d | (a)(b) |
| Cai 2012 | 2 | 2 | 53/45 | 43 vs 45 |  | 1 w~20 y | 55 vs 43 | (2) vs (3) | 10-22d | (a)(b) |
| Cao 2016 | 2 | 3 | N/A | 52.4 |  | N/A | 32 vs 32 | (1) vs (2) | 20d | (a)(b) |
| Chen 2000 | 2 | 3 | N/A | N/A |  | 3 d~7 y | 49 vs 50 | (1) vs (2) | 20d | (b) |
| Chen 2012 | 2 | 2 | 48/25 | N/A |  | 2 m~20 y | 37 vs 36 | (4) vs (3) | 20-90d | (a)(b) |
| Chen 2014 | 2 | 2 | 36/12 | N/A |  | N/A | 24 vs 24 | (1) vs (2) | 15-25d | (a)(b) |
| Dai 2015 | 2 | 2 | 100/80 | 45.9 vs 44.4 |  | 5.21±2.06 y vs 5.05±1.87 y | 90 vs 90 | (2) vs (3) | 20d | (a)(b) |
| Ding 2014 | 2 | 2 | 61/35 | N/A |  | 2.4±1.9 y | 48 vs 48 | (1) vs (2) | 14d | (a)(b)(d) |
| Dong 2010 | 2 | 2 | 101/94 | 43.7 vs 42.6 |  | 4.3±16.2 y vs 5.8±17.8 y | 99 vs 96 | (1) vs (2) | 24d | (a)(b)(c) |
| Duo 2016 | 2 | 2 | 150/130 | 45.6 vs 44.1 |  | 5.2±1.6 y vs 5.8±0.9 y | 140 vs 140 | (2) vs (3) | 20-45d | (a)(b)(c)(d) |
| Fan 2009 | 2 | 2 | 49/33 | N/A |  | 3 days~3 y | 45 vs 37 | (2) vs (3) | 20-30d | (a)(b)(c)(d) |
| Fang 2014 | 2 | 2 | 33/27 | 46.3 |  | 7.3±2.5 m | 30 vs 30 | (1) vs (2) | 21d | (a)(b) |
| Feng 2008 | 2 | 2 | 86/30 | 41 vs 42 |  | 3.1±1.8 m vs 3.3±1.5 m | 58 vs 58 | (4) vs (3) | 21d | (a)(b) |
| Fu 2011 | 2 | 3 | 148/127 | 37.9 vs 38.1 |  | 3.2±2.2 y vs 3.1±2.1 y | 139 vs 136 | (1) vs (2) | 42d | (a)(b) |
| Gao 2013 | 2 | 2 | 97/51 | 34.5 vs 33.1 |  | 10.1±0.5 m vs 11.2±0.6 m | 78 vs 70 | (2) vs (3) | 20d | (a)(b) |
| Geng 2008 | 2 | 2 | 102/70 | N/A |  | N/A | 122 vs 50 | (2) vs (3) | 20-30d | (a)(b) |
| Gong 2001 | 2 | 2 | 65/35 | N/A |  | N/A | 50 vs 50 | (1) vs (3) | 20d | (a)(b) |
| Gu 2011 | 2 | 2 | 30/30 | 40.8 vs 42.6 |  | 70.1±98. 9d vs90.2±121.8 d | 30 vs 30 | (1) vs (3) | 12d | (a)(b) |
| Gu 2013 | 2 | 3 | 68/52 | 41.9 vs 42.5 |  | 55.1±26.7d vs 70.2±32.2d | 60 vs 60 | (1) vs (3) | 12d | (a) |
| Guo 2014 | 2 | 3 | 21/23 | 52.5 vs 49.6 |  | 4.1±3.1 y vs 4.1±2. 6 y | 22 vs 22 | (1) vs (2) | 28d | (a)(b) |
| Han 2009 | 2 | 2 | 29/34 | N/A |  | N/A | 32 vs 31 | (1) vs (3) | 21d | (a)(b)(c)(d) |
| Hao 2017 | 2 | 2 | 107/73 | 51.2 vs 52.5 |  | 3.5±2.7 y vs 2.9±2.5 y | 90 vs 90 | (2) vs (3) | 90d | (a)(b)(c) |
| He 2011 | 2 | 2 | 32/28 | 40.2 vs 41.5 |  | 6.4±1.2 d vs 5.7±2.2 d | 30 vs 30 | (1) vs (3) | 28d | (a)(b)(c)(d) |
| He 2013 | 2 | 2 | 537/363 | N/A |  | N/A | 450 vs 450 | (2) vs (3) | 42d | (a)(b) |
| Hu 2014 | 2 | 2 | 44/38 | 45.7 |  | N/A | 41 vs 41 | (2) vs (3) | 28d | (a)(b)(c) |
| Hu 2015 | 2 | 3 | 26/50 | 48.3 vs 46.9 |  | 28.6±9.2 m vs 26.7±7.9 m | 38 vs 38 | (1) vs (4) | 14d | (a)(b) |
| Huang 2008 | 2 | 3 | 39/71 | 48.5 vs 49.1 |  | 12 d~21 y | 60 vs 50 | (1) vs (3) | 8d | (a)(b) |
| Huang 2013^a^ | 2 | 2 | 32/28 | 43 vs 47 |  | N/A | 30 vs 30 | (1) vs (2) | 20d | (c) |
| Huang 2013^b^ | 2 | 3 | 34/26 | 34.5 vs 35.7 |  | 21.5±14.9 d vs 20. 7±22.6 d | 30 vs 30 | (1) vs (2) | 14d | (a)(b) |
| Huang 2015 | 2 | 3 | N/A | N/A |  | N/A | 40 vs 40 | (1) vs (2) | 21d | (a)(b)(c) |
| Jia 2015 | 2 | 3 | 49/31 | 42.9 vs 44.3 |  | N/A | 40 vs 40 | (2) vs (4) | 14d | (a)(b) |
| Jiang 2005 | 2 | 2 | 37/23 | N/A |  | 3 d~7 y | 28 vs 32 | (4) vs (3) | 28d | (a)(b) |
| Jiang 2011 | 2 | 2 | 80/68 | N/A |  | N/A | 74 vs 74 | (1) vs (2) | 14d | (a)(b) |
| Jiang 2014 | 2 | 2 | 56/44 | 42.6 vs 42. 6 |  | 5.7±1.4 y vs 5. 7±1.4 y | 50 vs 50 | (2) vs (3) | N/A | (a)(b) |
| Jiao 2005 | 2 | 3 | N/A | N/A |  | N/A | 86 vs 30 | (3) vs (4) | 21d | (a)(b) |
| Ju 2014 | 2 | 2 | 100/46 | 40.15 |  | 2 m~6 y | 80 vs 66 | (1) vs (3) | 30d | (a)(b)(c) |
| Lei 2018 | 2 | 3 | 56/64 | 34 vs 35 |  | 235.6±30.2d vs215.3±32.6d | 60 vs 60 | (1) vs (2) | 20d | (a)(b)(c)(d) |
| Li 2003 | 2 | 2 | 21/29 | N/A |  | 23.9±5.7 | 30 vs 20 | (1) vs (3) | 28d | (a)(b)(c) |
| Li 2010 | 2 | 2 | 65/35 | N/A |  | 7 d~10 y | 53 vs 47 | (1) vs (2) | 28d | (a)(b) |
| Li 2012 | 2 | 2 | 37/23 | N/A |  | 3 d~7 y | 28 vs 32 | (4) vs (3) | 28d | (a)(b) |
| Li 2013 | 2 | 2 | 48/52 | N/A |  | N/A | 50 vs 50 | (1) vs (4) | 30d | (a)(b) |
| Li 2014 | 2 | 2 | 48/42 | N/A |  | N/A | 45 vs 45 | (2) vs (3) | 35d | (a)(b)(d) |
| Li 2016 | 2 | 2 | 53/27 | 29.1 vs 30 |  | 0.5±0.3 y | 40 vs 40 | (1) vs (4) | 30d | (a)(b) |
| Liang 2015 | 2 | 3 | N/A | 44.9 vs 42.3 |  | N/A | 29 vs 29 | (1) vs (2) | 28d | (a)(b) |
| Liao 2010 | 2 | 3 | N/A | 39.2 |  | 12.5±1.6 m | 45 vs 45 | (2) vs (4) | 20d | (a)(b) |
| Lin 2013 | 2 | 2 | 58/44 | 39.2 vs 38.9 |  | N/A | 51 vs 51 | (2) vs (3) | 20d | (a)(b) |
| Liu 2008 | 2 | 2 | 37/33 | N/A |  | N/A | 35 vs 35 | (1) vs (3) | 10d | (a)(b)(c) |
| Liu 2009 | 2 | 3 | 47/30 | 38.9 vs 36.3 |  | 15.3±14.0m vs 14.3±13.9m | 42 vs 35 | (2) vs (4) | 20d | (a)(b) |
| Liu 2012 | 2 | 2 | 80/72 | 39 vs 38 |  | 8.3±2.7 m vs 8.1±2.3 m | 76 vs 76 | (1) vs (2) | 15d | (a)(b) |
| Liu 2013^a^ | 3 | 5 | N/A | N/A |  | N/A | 30vs30vs30 | (1) vs (2) vs (4) | 30d | (a)(b) |
| Liu 2013^b^ | 2 | 2 | 43/24 | N/A |  | N/A | 33 vs 34 | (1) vs (2) | 21d | (a)(b) |
| Liu 2014 | 2 | 2 | 34/22 | 47.1 |  | N/A | 28 vs 28 | (2) vs (3) | 10d | (a)(b) |
| Liu 2016 | 2 | 2 | 44/46 | 44.2 vs 43.2 |  | N/A | 45 vs 45 | (3) vs (4) | 30d | (c)(d) |
| Lun 2006 | 2 | 2 | 87/63 | N/A |  | N/A | 75 vs 75 | (1) vs (3) | 60d | (a)(b)(d) |
| Luo 2008 | 2 | 2 | 37/29 | 39.3 vs 40.7 |  | 2.1±1.2 y vs 2.2±1.3 y | 33 vs 33 | (2) vs (3) | 25d | (a)(b) |
| Luo 2009 | 2 | 2 | 47/33 | 47.2 vs 48.1 |  | 21.1±3.1 m vs 20.3±3.5 m | 40 vs 40 | (3) vs (4) | 20-40d | (a)(b)(c) |
| Ma 2016 | 2 | 2 | 47/53 | N/A |  | N/A | 50 vs 50 | (2) vs (4) | 15d | (a)(b) |
| Mao 2009 | 2 | 2 | 85/75 | N/A |  | N/A | 80 vs 80 | (1) vs (3) | 14d | (a)(b) |
| Meng 2010 | 2 | 3 | 72/48 | 44.3 vs 41.0 |  | 2.7±1.8 y vs 2.3±0.14 y | 60 vs 60 | (1) vs (4) | 21d | (a)(b) |
| Meng 2013 | 2 | 2 | 96/104 | N/A |  | N/A | 100 vs 100 | (1) vs (4) | 30d | (a)(b) |
| Ning 2015 | 2 | 2 | 21/39 | 41.1 vs 43.1 |  | N/A | 30 vs 30 | (1) vs (3) | 14d | (c)(d) |
| Quan 2006 | 2 | 2 | 43/37 | N/A |  | N/A | 40 vs 40 | (1) vs (3) | 28d | (a)(b) |
| Sha 2011 | 2 | 2 | 128/108 | N/A |  | N/A | 118 vs 118 | (1) vs (3) | 30d | (a)(b) |
| Sha 2017 | 2 | 2 | 18/14 | 42.9 vs 43.5 |  | N/A | 16 vs 16 | (1) vs (3) | 30d | (a)(b)(c) |
| She 2016 | 2 | 2 | N/A | N/A |  | N/A | 184 vs 176 | (1) vs (2) | 14d | (a)(b) |
| Shen 2016 | 2 | 2 | 164/106 | N/A |  | N/A | 135 vs 135 | (1) vs (3) | 14d | (a)(b)(d) |
| Shi 2010 | 2 | 2 | 32/38 | N/A |  | N/A | 35 vs 35 | (1) vs (3) | 7d | (a)(b) |
| Song 2005 | 2 | 2 | 71/47 | N/A |  | N/A | 58 vs 60 | (1) vs (3) | 21d | (a)(b) |
| Song 2010 | 2 | 2 | 42/18 | N/A |  | N/A | 30 vs 30 | (1) vs (4) | 30d | (a)(b) |
| Su 2013 | 2 | 3 | 26/34 | 52.6 vs 48.8 |  | 12.6±12.9m vs 12.3±16.1 m | 30 vs 30 | (1) vs (2) | 14d | (a)(b) |
| Sun 2012 | 2 | 2 | 40/20 | N/A |  | N/A | 30 vs 30 | (2) vs (3) | 28d | (a)(b)(c)(d) |
| Sun 2014^a^ | 2 | 2 | 41/29 | 41.4 vs 42.1 |  | N/A | 35 vs 35 | (2) vs (3) | 10-15d | (a)(b)(c)(d) |
| Sun 2014^b^ | 2 | 4 | 19/21 | 45.3 vs 44.9 |  | N/A | 20 vs 20 | (2) vs (3) | 21d | (a)(b) |
| Sun 2016 | 2 | 2 | 67/53 | 45.7 vs 44 |  | 6.8±2.3 m vs 6.5±3.1m | 60 vs 60 | (1) vs (2) | 10d | (a)(b) |
| Tang 2009 | 2 | 2 | 51/49 | 42.5 vs 43.4 |  | N/A | 50 vs 50 | (2) vs (3) | 15d | (a)(b) |
| Tang 2012 | 2 | 2 | 50/45 | N/A |  | N/A | 48 vs 47 | (1) vs (3) | 10d | (a)(b)(c) |
| Tu 2014 | 2 | 2 | 70/30 | N/A |  | N/A | 60 vs 40 | (1) vs (4) | 10-20d | (a)(b) |
| Wang 2007 | 2 | 3 | 40/31 | N/A |  | N/A | 34 vs 37 | (1) vs (3) | 30d | (a)(b) |
| Wang 2010 | 2 | 2 | 37/25 | N/A |  | N/A | 32 vs 30 | (1) vs (3) | 21d | (a)(b)(c)(d) |
| Wang 2013 | 2 | 2 | 31/99 | N/A |  | N/A | 65 vs 65 | (1) vs (3) | 21d | (a)(b) |
| Wang 2016 | 2 | 2 | 35/25 | 51.3 |  | 3.5±0.9 m | 30 vs 30 | (1) vs (3) | 14d | (a)(b) |
| Wang 2017 | 2 | 2 | 38/26 | N/A |  | N/A | 32 vs 32 | (1) vs (3) | 20d | (a)(b)(c)(d) |
| Wen 2010 | 2 | 3 | N/A | N/A |  | N/A | 40 vs 40 | (1) vs (4) | 21d | (a)(b)(c) |
| Wu 2007 | 2 | 3 | N/A | N/A |  | N/A | 70 vs 85 | (1) vs (3) | 20d | (a)(b) |
| Wu 2011 | 2 | 3 | N/A | 36.6 vs 39.2 |  | N/A | 33 vs 33 | (1) vs (2) | 21d | (a)(b) |
| Wu 2015^a^ | 2 | 2 | 33/27 | 46.7 vs 46.5 |  | 15.2±3.5 d vs 16.2±2.5 d | 30 vs 30 | (2) vs (3) | 20-30d | (a)(b)(c)(d) |
| Wu 2015^b^ | 2 | 2 | 25/17 | 42.8 vs 43.6 |  | 17.9±3.8 d vs 18.1±2.4 d | 21 vs 21 | (2) vs (3) | 20d | (d) |
| Xie 2009 | 2 | 2 | 36/24 | N/A |  | N/A | 30 vs 30 | (1) vs (3) | 21d | (a)(b) |
| Xie 2011 | 2 | 2 | 245/115 | N/A |  | N/A | 180 vs 180 | (2) vs (3) | 28d | (a)(b) |
| Xiong 2013 | 2 | 3 | 43/17 | 44.4 vs 44.2 |  | 1.5±0.4 y vs 1.4±0.5 y | 30 vs 30 | (1) vs (2) | 30d | (a)(b) |
| Xiong 2016 | 2 | 2 | 47/41 | N/A |  | N/A | 44 vs 44 | (2) vs (3) | 30d | (a)(b) |
| Xuan 2013 | 2 | 2 | 34/26 | 38.5 |  | N/A | 30 vs 30 | (3) vs (4) | 14-21d | (a)(b) |
| Xue 2017 | 2 | 2 | 62/38 | 42.1 vs 42.6 |  | N/A | 50 vs 50 | (1) vs (3) | 30d | (a)(b)(c)(d) |
| Yang 2004 | 2 | 3 | 46/25 | 44.5 vs 40.9 |  | N/A | 38 vs 33 | (1) vs (4) | 15d | (a)(b) |
| Yang 2006 | 2 | 2 | N/A | N/A |  | N/A | 32 vs 28 | (2) vs (4) | 10d | (a)(b) |
| Yang 2010 | 2 | 2 | 30/30 | N/A |  | N/A | 30 vs 30 | (1) vs (3) | 10d | (a)(b)(c)(d) |
| Yang 2014 | 2 | 2 | 54/46 | 41 vs 40.5 |  | 14.2±3.4 m vs 14.7±3.8 m | 50 vs 50 | (1) vs (2) | 10d | (a)(b)(d) |
| Yang 2016^a^ | 2 | 2 | 40/60 | 48.9 vs 48.2 |  | N/A | 50 vs 50 | (1) vs (2) | 28d | (a)(b)(c) |
| Yang 2016^b^ | 2 | 3 | 37/29 | N/A |  | N/A | 33 vs 33 | (1) vs (2) | 28d | (a)(b) |
| Ye 2012 | 2 | 3 | 53/67 | 40.1 vs 38.9 |  | 9.1±8.9 m vs 9.2±9.1 m | 60 vs 60 | (2) vs (3) | 10d | (a)(b) |
| Yu 2008^a^ | 2 | 2 | 63/61 | N/A |  | N/A | 62 vs 62 | (1) vs (4) | 14d | (a)(b) |
| Yu 2008^b^ | 2 | 2 | 53/51 | N/A |  | N/A | 52 vs 52 | (1) vs (3) | 14d | (a)(b) |
| Yun 2014 | 3 | 4 | 24/24 | N/A |  | N/A | 16 vs 16 vs 16 | (1) vs (2) vs (4) | 20-30d | (a)(b) |
| Zhan 2017 | 2 | 2 | 26/42 | 48.3 vs 47.7 |  | 4.3±1. 8 y vs 4.4±2.1 y | 34 vs 34 | (2) vs (4) | 20d | (a)(b)(c) |
| Zhang 2002 | 2 | 3 | 116/66 | N/A |  | N/A | 98 vs 84 | (1) vs (2) | 10d | (a)(b) |
| Zhang 2010 | 2 | 2 | 29/34 | 36.2 vs 37.1 |  | 6.9±2.5 y vs 6.2±2.9 y | 32 vs 31 | (1) vs (3) | 21d | (a)(b)(c)(d) |
| Zhang 2012 | 2 | 2 | 56/46 | N/A |  | N/A | 50 vs 50 | (3) vs (4) | 14d | (a)(b) |
| Zhang 2014 | 2 | 3 | 48/52 | 42.2 vs 41.2 |  | N/A | 50 vs 50 | (1) vs (2) | N/A | (a)(b) |
| Zhang 2015 | 2 | 3 | 26/12 | 42.4 vs 35.6 |  | N/A | 19 vs 19 | (1) vs (2) | 14d | (a)(b) |
| Zhang 2016^a^ | 2 | 2 | 72/28 | 51.4 vs 51.8 |  | 14.3±5.8d vs 14.2±5.6 d | 50 vs 50 | (2) vs (3) | 28d | (a)(b)(c) |
| Zhang 2016^b^ | 2 | 2 | N/A | N/A |  | N/A | 40 vs 40 | (1) vs (2) | 14d | (a)(b) |
| Zhang 2016^c^ | 2 | 3 | 24/16 | 50.1 vs 48.1 |  | 3.2±1.8 y vs 2.6±1.5 y | 20 vs 20 | (2) vs (4) | 28d | (a)(b) |
| Zhang 2017 | 2 | 2 | 29/31 | 42.3 vs 44.02 |  | 16.7±2.5 m vs 18.0±3.2 m | 30 vs 30 | (2) vs (3) | N/A | (c) |
| Zhao 2007^a^ | 2 | 2 | 68/48 | N/A |  | N/A | 58 vs 58 | (1) vs (3) | 30d | (a)(b) |
| Zhao 2007^b^ | 2 | 2 | 90/58 | N/A |  | N/A | 85 vs 63 | (1) vs (3) | 20d | (a)(b) |
| Zhao 2008 | 2 | 3 | N/A | N/A |  | N/A | 36 vs 36 | (2) vs (3) | 14d | (a)(b)(c) |
| Zhao 2011 | 2 | 3 | 31/29 | N/A |  | N/A | 30 vs 30 | (2) vs (4) | 20d | (a)(b) |
| Zhi 2001 | 3 | 4 | 74/61 | N/A |  | 6.8±1.86yvs7.5±1.83yvs8.1±1.78y | 45 vs 45 vs 45 | (1) vs (2) vs (3) | 30d | (a)(b) |
| Zhou 2001 | 2 | 3 | N/A | N/A |  | N/A | 90 vs 87 | (1) vs (3) | 30d | (a)(b) |
| Zhou 2010 | 2 | 3 | N/A | N/A |  | N/A | 47 vs 58 | (2) vs (4) | 30d | (a)(b) |
| Zhu 2012 | 2 | 3 | N/A | 39.18 |  | 12.5±1.6 m | 45 vs 45 | (2) vs (4) | 20d | (a)(b) |

Note: (1) =Tuina, (2) =Acupuncture, (3) =Traction, (4) =Chinese herbs, (a) =invalid rate, (b)=cure rate, (c)=VAS, (d)=JOA, vs=versus, d=day, w=week, m=month, y=year, m/f=male/female n=number

## 4 Risk of bias of included studies

| Study ID | Sequence generation | Allocation concealment | Blinding (participant) | Blinding  (therapist) | Blinding  (assessor) | Attrition bias | reporting bias |
| --- | --- | --- | --- | --- | --- | --- | --- |
| Ao 2008 | low risk of bias | unclear risk of bias | unclear risk of bias | unclear risk of bias | unclear risk of bias | low risk of bias | unclear risk of bias |
| Cai 2012 | unclear risk of bias | unclear risk of bias | unclear risk of bias | unclear risk of bias | unclear risk of bias | low risk of bias | unclear risk of bias |
| Cao 2016 | unclear risk of bias | unclear risk of bias | unclear risk of bias | unclear risk of bias | unclear risk of bias | low risk of bias | unclear risk of bias |
| Chen 2000 | unclear risk of bias | unclear risk of bias | unclear risk of bias | unclear risk of bias | unclear risk of bias | low risk of bias | unclear risk of bias |
| Chen 2012 | unclear risk of bias | unclear risk of bias | unclear risk of bias | unclear risk of bias | unclear risk of bias | low risk of bias | unclear risk of bias |
| Chen 2014 | unclear risk of bias | unclear risk of bias | unclear risk of bias | unclear risk of bias | unclear risk of bias | low risk of bias | unclear risk of bias |
| Dai 2015 | unclear risk of bias | unclear risk of bias | unclear risk of bias | unclear risk of bias | unclear risk of bias | low risk of bias | unclear risk of bias |
| Ding 2014 | unclear risk of bias | unclear risk of bias | unclear risk of bias | unclear risk of bias | unclear risk of bias | low risk of bias | unclear risk of bias |
| Dong 2010 | unclear risk of bias | unclear risk of bias | unclear risk of bias | unclear risk of bias | unclear risk of bias | low risk of bias | unclear risk of bias |
| Duo 2016 | low risk of bias | unclear risk of bias | unclear risk of bias | unclear risk of bias | unclear risk of bias | low risk of bias | unclear risk of bias |
| Fan 2009 | low risk of bias | unclear risk of bias | unclear risk of bias | unclear risk of bias | unclear risk of bias | low risk of bias | unclear risk of bias |
| Fang 2014 | unclear risk of bias | unclear risk of bias | unclear risk of bias | unclear risk of bias | unclear risk of bias | low risk of bias | unclear risk of bias |
| Feng 2008 | unclear risk of bias | unclear risk of bias | unclear risk of bias | unclear risk of bias | unclear risk of bias | low risk of bias | unclear risk of bias |
| Fu 2011 | unclear risk of bias | unclear risk of bias | unclear risk of bias | unclear risk of bias | unclear risk of bias | low risk of bias | unclear risk of bias |
| Gao 2013 | low risk of bias | unclear risk of bias | unclear risk of bias | unclear risk of bias | unclear risk of bias | low risk of bias | unclear risk of bias |
| Geng2008 | unclear risk of bias | unclear risk of bias | unclear risk of bias | unclear risk of bias | unclear risk of bias | low risk of bias | unclear risk of bias |
| Gong 2001 | unclear risk of bias | unclear risk of bias | unclear risk of bias | unclear risk of bias | unclear risk of bias | low risk of bias | unclear risk of bias |
| Gu 2011 | unclear risk of bias | unclear risk of bias | unclear risk of bias | unclear risk of bias | unclear risk of bias | low risk of bias | unclear risk of bias |
| Gu 2013 | unclear risk of bias | unclear risk of bias | unclear risk of bias | unclear risk of bias | unclear risk of bias | low risk of bias | unclear risk of bias |
| Guo 2014 | unclear risk of bias | unclear risk of bias | unclear risk of bias | unclear risk of bias | unclear risk of bias | low risk of bias | unclear risk of bias |
| Han 2009 | low risk of bias | low risk of bias | unclear risk of bias | unclear risk of bias | unclear risk of bias | low risk of bias | unclear risk of bias |
| Hao 2017 | unclear risk of bias | unclear risk of bias | unclear risk of bias | unclear risk of bias | unclear risk of bias | low risk of bias | unclear risk of bias |
| He 2011 | unclear risk of bias | unclear risk of bias | unclear risk of bias | unclear risk of bias | unclear risk of bias | low risk of bias | unclear risk of bias |
| He 2013 | unclear risk of bias | unclear risk of bias | unclear risk of bias | unclear risk of bias | unclear risk of bias | low risk of bias | unclear risk of bias |
| Hu 2014 | unclear risk of bias | unclear risk of bias | unclear risk of bias | unclear risk of bias | unclear risk of bias | low risk of bias | unclear risk of bias |
| Hu 2015 | low risk of bias | unclear risk of bias | unclear risk of bias | unclear risk of bias | unclear risk of bias | low risk of bias | unclear risk of bias |
| Huang 2008 | unclear risk of bias | unclear risk of bias | unclear risk of bias | unclear risk of bias | unclear risk of bias | low risk of bias | unclear risk of bias |
| Huang 2013^a^ | unclear risk of bias | unclear risk of bias | unclear risk of bias | unclear risk of bias | unclear risk of bias | low risk of bias | unclear risk of bias |
| Huang 2013^b^ | low risk of bias | unclear risk of bias | unclear risk of bias | unclear risk of bias | unclear risk of bias | low risk of bias | unclear risk of bias |
| Huang 2015 | unclear risk of bias | unclear risk of bias | unclear risk of bias | unclear risk of bias | unclear risk of bias | low risk of bias | unclear risk of bias |
| Jia 2015 | low risk of bias | unclear risk of bias | unclear risk of bias | unclear risk of bias | unclear risk of bias | low risk of bias | unclear risk of bias |
| Jiang 2005 | unclear risk of bias | unclear risk of bias | unclear risk of bias | unclear risk of bias | unclear risk of bias | low risk of bias | unclear risk of bias |
| Jiang 2011 | unclear risk of bias | unclear risk of bias | unclear risk of bias | unclear risk of bias | unclear risk of bias | low risk of bias | unclear risk of bias |
| Jiang 2014 | unclear risk of bias | unclear risk of bias | unclear risk of bias | unclear risk of bias | unclear risk of bias | low risk of bias | unclear risk of bias |
| Jiao 2005 | unclear risk of bias | unclear risk of bias | unclear risk of bias | unclear risk of bias | unclear risk of bias | low risk of bias | unclear risk of bias |
| Ju 2014 | unclear risk of bias | unclear risk of bias | unclear risk of bias | unclear risk of bias | unclear risk of bias | low risk of bias | unclear risk of bias |
| Lei 2018 | unclear risk of bias | unclear risk of bias | unclear risk of bias | unclear risk of bias | unclear risk of bias | low risk of bias | unclear risk of bias |
| Li 2003 | unclear risk of bias | unclear risk of bias | unclear risk of bias | unclear risk of bias | unclear risk of bias | low risk of bias | unclear risk of bias |
| Li 2010 | unclear risk of bias | unclear risk of bias | unclear risk of bias | unclear risk of bias | unclear risk of bias | low risk of bias | unclear risk of bias |
| Li 2012 | unclear risk of bias | unclear risk of bias | unclear risk of bias | unclear risk of bias | unclear risk of bias | low risk of bias | unclear risk of bias |
| Li 2013 | unclear risk of bias | unclear risk of bias | unclear risk of bias | unclear risk of bias | unclear risk of bias | low risk of bias | unclear risk of bias |
| Li 2014 | low risk of bias | unclear risk of bias | unclear risk of bias | unclear risk of bias | unclear risk of bias | low risk of bias | unclear risk of bias |
| Li 2016 | unclear risk of bias | unclear risk of bias | unclear risk of bias | unclear risk of bias | unclear risk of bias | low risk of bias | unclear risk of bias |
| Liang 2015 | unclear risk of bias | unclear risk of bias | unclear risk of bias | unclear risk of bias | unclear risk of bias | low risk of bias | unclear risk of bias |
| Liao 2010 | low risk of bias | low risk of bias | unclear risk of bias | unclear risk of bias | unclear risk of bias | low risk of bias | unclear risk of bias |
| Lin 2013 | low risk of bias | unclear risk of bias | unclear risk of bias | unclear risk of bias | unclear risk of bias | low risk of bias | unclear risk of bias |
| Liu 2008 | unclear risk of bias | unclear risk of bias | unclear risk of bias | unclear risk of bias | unclear risk of bias | low risk of bias | unclear risk of bias |
| Liu 2009 | unclear risk of bias | unclear risk of bias | unclear risk of bias | unclear risk of bias | unclear risk of bias | low risk of bias | unclear risk of bias |
| Liu 2012 | unclear risk of bias | unclear risk of bias | unclear risk of bias | unclear risk of bias | unclear risk of bias | low risk of bias | unclear risk of bias |
| Liu 2013^a^ | unclear risk of bias | unclear risk of bias | unclear risk of bias | unclear risk of bias | unclear risk of bias | low risk of bias | unclear risk of bias |
| Liu 2013^b^ | unclear risk of bias | unclear risk of bias | unclear risk of bias | unclear risk of bias | unclear risk of bias | low risk of bias | unclear risk of bias |
| Liu 2014 | unclear risk of bias | unclear risk of bias | unclear risk of bias | unclear risk of bias | unclear risk of bias | low risk of bias | unclear risk of bias |
| Liu 2016 | unclear risk of bias | unclear risk of bias | unclear risk of bias | unclear risk of bias | unclear risk of bias | low risk of bias | unclear risk of bias |
| Lun 2006 | unclear risk of bias | unclear risk of bias | unclear risk of bias | unclear risk of bias | unclear risk of bias | low risk of bias | unclear risk of bias |
| Luo 2008 | unclear risk of bias | unclear risk of bias | unclear risk of bias | unclear risk of bias | unclear risk of bias | low risk of bias | unclear risk of bias |
| Luo 2009 | low risk of bias | unclear risk of bias | unclear risk of bias | unclear risk of bias | unclear risk of bias | low risk of bias | unclear risk of bias |
| Ma 2016 | low risk of bias | unclear risk of bias | unclear risk of bias | unclear risk of bias | unclear risk of bias | low risk of bias | unclear risk of bias |
| Mao 2009 | unclear risk of bias | unclear risk of bias | unclear risk of bias | unclear risk of bias | unclear risk of bias | low risk of bias | unclear risk of bias |
| Meng 2010 | unclear risk of bias | unclear risk of bias | unclear risk of bias | unclear risk of bias | unclear risk of bias | low risk of bias | unclear risk of bias |
| Meng 2013 | unclear risk of bias | unclear risk of bias | unclear risk of bias | unclear risk of bias | unclear risk of bias | low risk of bias | unclear risk of bias |
| Ning 2015 | unclear risk of bias | unclear risk of bias | unclear risk of bias | unclear risk of bias | unclear risk of bias | low risk of bias | unclear risk of bias |
| Quan 2006 | unclear risk of bias | unclear risk of bias | unclear risk of bias | unclear risk of bias | unclear risk of bias | low risk of bias | unclear risk of bias |
| Sha 2011 | unclear risk of bias | unclear risk of bias | unclear risk of bias | unclear risk of bias | unclear risk of bias | low risk of bias | unclear risk of bias |
| Sha 2017 | unclear risk of bias | unclear risk of bias | unclear risk of bias | unclear risk of bias | unclear risk of bias | low risk of bias | unclear risk of bias |
| She 2016 | low risk of bias | unclear risk of bias | unclear risk of bias | unclear risk of bias | unclear risk of bias | low risk of bias | unclear risk of bias |
| Shen 2016 | low risk of bias | unclear risk of bias | unclear risk of bias | unclear risk of bias | unclear risk of bias | low risk of bias | unclear risk of bias |
| Shi 2010 | unclear risk of bias | unclear risk of bias | unclear risk of bias | unclear risk of bias | unclear risk of bias | low risk of bias | unclear risk of bias |
| Song 2005 | low risk of bias | unclear risk of bias | unclear risk of bias | unclear risk of bias | unclear risk of bias | low risk of bias | unclear risk of bias |
| Song 2010 | unclear risk of bias | unclear risk of bias | unclear risk of bias | unclear risk of bias | unclear risk of bias | low risk of bias | unclear risk of bias |
| Su 2013 | unclear risk of bias | unclear risk of bias | unclear risk of bias | unclear risk of bias | unclear risk of bias | low risk of bias | unclear risk of bias |
| Sun 2012 | low risk of bias | unclear risk of bias | unclear risk of bias | unclear risk of bias | unclear risk of bias | low risk of bias | unclear risk of bias |
| Sun 2014^a^ | low risk of bias | unclear risk of bias | unclear risk of bias | unclear risk of bias | unclear risk of bias | low risk of bias | unclear risk of bias |
| Sun 2014^b^ | unclear risk of bias | unclear risk of bias | unclear risk of bias | unclear risk of bias | unclear risk of bias | low risk of bias | unclear risk of bias |
| Sun 2016 | unclear risk of bias | unclear risk of bias | unclear risk of bias | unclear risk of bias | unclear risk of bias | low risk of bias | unclear risk of bias |
| Tang 2009 | unclear risk of bias | unclear risk of bias | unclear risk of bias | unclear risk of bias | unclear risk of bias | low risk of bias | unclear risk of bias |
| Tang 2012 | unclear risk of bias | unclear risk of bias | unclear risk of bias | unclear risk of bias | unclear risk of bias | low risk of bias | unclear risk of bias |
| Tu 2014 | unclear risk of bias | unclear risk of bias | unclear risk of bias | unclear risk of bias | unclear risk of bias | low risk of bias | unclear risk of bias |
| Wang 2007 | unclear risk of bias | unclear risk of bias | unclear risk of bias | unclear risk of bias | unclear risk of bias | low risk of bias | unclear risk of bias |
| Wang 2010 | low risk of bias | low risk of bias | unclear risk of bias | unclear risk of bias | unclear risk of bias | low risk of bias | unclear risk of bias |
| Wang 2013 | unclear risk of bias | unclear risk of bias | unclear risk of bias | unclear risk of bias | unclear risk of bias | low risk of bias | unclear risk of bias |
| Wang 2016 | unclear risk of bias | unclear risk of bias | unclear risk of bias | unclear risk of bias | unclear risk of bias | low risk of bias | unclear risk of bias |
| Wang 2017 | unclear risk of bias | unclear risk of bias | unclear risk of bias | unclear risk of bias | unclear risk of bias | low risk of bias | unclear risk of bias |
| Wen 2010 | unclear risk of bias | unclear risk of bias | unclear risk of bias | unclear risk of bias | unclear risk of bias | low risk of bias | unclear risk of bias |
| Wu 2007 | unclear risk of bias | unclear risk of bias | unclear risk of bias | unclear risk of bias | unclear risk of bias | low risk of bias | unclear risk of bias |
| Wu 2011 | low risk of bias | low risk of bias | unclear risk of bias | unclear risk of bias | unclear risk of bias | low risk of bias | unclear risk of bias |
| Wu 2015^a^ | unclear risk of bias | unclear risk of bias | unclear risk of bias | unclear risk of bias | unclear risk of bias | low risk of bias | unclear risk of bias |
| Wu 2015^b^ | unclear risk of bias | unclear risk of bias | unclear risk of bias | unclear risk of bias | unclear risk of bias | low risk of bias | unclear risk of bias |
| Xie 2009 | unclear risk of bias | unclear risk of bias | unclear risk of bias | unclear risk of bias | unclear risk of bias | low risk of bias | unclear risk of bias |
| Xie 2011 | unclear risk of bias | unclear risk of bias | unclear risk of bias | unclear risk of bias | unclear risk of bias | low risk of bias | unclear risk of bias |
| Xiong 2013 | unclear risk of bias | unclear risk of bias | unclear risk of bias | unclear risk of bias | unclear risk of bias | low risk of bias | unclear risk of bias |
| Xiong 2016 | unclear risk of bias | unclear risk of bias | unclear risk of bias | unclear risk of bias | unclear risk of bias | low risk of bias | unclear risk of bias |
| Xuan 2013 | unclear risk of bias | unclear risk of bias | unclear risk of bias | unclear risk of bias | unclear risk of bias | low risk of bias | unclear risk of bias |
| Xue 2017 | unclear risk of bias | unclear risk of bias | unclear risk of bias | unclear risk of bias | unclear risk of bias | low risk of bias | unclear risk of bias |
| Yang 2004 | unclear risk of bias | unclear risk of bias | unclear risk of bias | unclear risk of bias | unclear risk of bias | low risk of bias | unclear risk of bias |
| Yang 2006 | unclear risk of bias | unclear risk of bias | unclear risk of bias | unclear risk of bias | unclear risk of bias | low risk of bias | unclear risk of bias |
| Yang 2010 | unclear risk of bias | unclear risk of bias | unclear risk of bias | unclear risk of bias | unclear risk of bias | low risk of bias | unclear risk of bias |
| Yang 2014 | unclear risk of bias | unclear risk of bias | unclear risk of bias | unclear risk of bias | unclear risk of bias | low risk of bias | unclear risk of bias |
| Yang 2016^a^ | low risk of bias | unclear risk of bias | unclear risk of bias | unclear risk of bias | unclear risk of bias | low risk of bias | unclear risk of bias |
| Yang 2016^b^ | unclear risk of bias | unclear risk of bias | unclear risk of bias | unclear risk of bias | unclear risk of bias | low risk of bias | unclear risk of bias |
| Ye 2012 | unclear risk of bias | unclear risk of bias | unclear risk of bias | unclear risk of bias | unclear risk of bias | low risk of bias | unclear risk of bias |
| Yu 2008^a^ | unclear risk of bias | unclear risk of bias | unclear risk of bias | unclear risk of bias | unclear risk of bias | low risk of bias | unclear risk of bias |
| Yu 2008^b^ | unclear risk of bias | unclear risk of bias | unclear risk of bias | unclear risk of bias | unclear risk of bias | low risk of bias | unclear risk of bias |
| Yun 2014 | unclear risk of bias | unclear risk of bias | unclear risk of bias | unclear risk of bias | unclear risk of bias | low risk of bias | unclear risk of bias |
| Zhan 2017 | low risk of bias | unclear risk of bias | unclear risk of bias | unclear risk of bias | unclear risk of bias | low risk of bias | unclear risk of bias |
| Zhang 2002 | unclear risk of bias | unclear risk of bias | unclear risk of bias | unclear risk of bias | unclear risk of bias | low risk of bias | unclear risk of bias |
| Zhang 2010 | low risk of bias | low risk of bias | unclear risk of bias | unclear risk of bias | unclear risk of bias | low risk of bias | unclear risk of bias |
| Zhang 2012 | unclear risk of bias | unclear risk of bias | unclear risk of bias | unclear risk of bias | unclear risk of bias | low risk of bias | unclear risk of bias |
| Zhang 2014 | unclear risk of bias | unclear risk of bias | unclear risk of bias | unclear risk of bias | unclear risk of bias | low risk of bias | unclear risk of bias |
| Zhang 2015 | unclear risk of bias | unclear risk of bias | unclear risk of bias | unclear risk of bias | unclear risk of bias | low risk of bias | unclear risk of bias |
| Zhang 2016^a^ | low risk of bias | unclear risk of bias | unclear risk of bias | unclear risk of bias | unclear risk of bias | low risk of bias | unclear risk of bias |
| Zhang 2016^b^ | unclear risk of bias | unclear risk of bias | unclear risk of bias | unclear risk of bias | unclear risk of bias | low risk of bias | unclear risk of bias |
| Zhang 2016^c^ | low risk of bias | unclear risk of bias | unclear risk of bias | unclear risk of bias | unclear risk of bias | low risk of bias | unclear risk of bias |
| Zhang 2017 | low risk of bias | unclear risk of bias | unclear risk of bias | unclear risk of bias | unclear risk of bias | low risk of bias | unclear risk of bias |
| Zhao 2007^a^ | low risk of bias | unclear risk of bias | unclear risk of bias | unclear risk of bias | unclear risk of bias | low risk of bias | unclear risk of bias |
| Zhao 2007^b^ | unclear risk of bias | unclear risk of bias | unclear risk of bias | unclear risk of bias | unclear risk of bias | low risk of bias | unclear risk of bias |
| Zhao 2008 | unclear risk of bias | unclear risk of bias | unclear risk of bias | unclear risk of bias | unclear risk of bias | low risk of bias | unclear risk of bias |
| Zhao 2011 | unclear risk of bias | unclear risk of bias | unclear risk of bias | unclear risk of bias | unclear risk of bias | low risk of bias | unclear risk of bias |
| Zhi 2001 | low risk of bias | unclear risk of bias | unclear risk of bias | unclear risk of bias | unclear risk of bias | low risk of bias | unclear risk of bias |
| Zhou 2001 | unclear risk of bias | unclear risk of bias | unclear risk of bias | unclear risk of bias | unclear risk of bias | low risk of bias | unclear risk of bias |
| Zhou 2010 | unclear risk of bias | unclear risk of bias | unclear risk of bias | unclear risk of bias | unclear risk of bias | low risk of bias | unclear risk of bias |
| Zhu 2012 | unclear risk of bias | unclear risk of bias | unclear risk of bias | unclear risk of bias | unclear risk of bias | low risk of bias | unclear risk of bias |

## 5 Node-splitting

5.1 Invalid rate

| Side | Direct | | Indirect | | Difference | | P>\|z\| |
| --- | --- | --- | --- | --- | --- | --- | --- |
|  | Coef. | Std. Err. | Coef. | Std. Err. | Coef. | Std. Err. |  |
| C D | -.8277416 | .320967 | -.5855057 | .2025279 | -.2422359 | .3796016 | 0.523 |
| A C | .9887651 | .1475772 | 1.303563 | .1965582 | -.3147981 | .2454073 | 0.200 |
| A B | -.0752937 | .1619702 | -.0276869 | .1858546 | -.0476069 | .2465601 | 0.847 |
| A D | -.7775962 | .2351119 | .1517603 | .2211123 | .6258358 | .3218664 | 0.052 |
| B C | 1.249387 | .1622561 | 1.030943 | .1913109 | .2184436 | .250801 | 0.384 |
| B D | .2536336 | .2411279 | .7128706 | .22156 | -.459237 | .3272474 | 0.161 |

Note: A=Tuina, B=Acupuncture, C=Traction, D=Chinese herbs

5.2 Cure rate

| Side | Direct | | Indirect | | Difference | | P>\|z\| |
| --- | --- | --- | --- | --- | --- | --- | --- |
|  | Coef. | Std. Err. | Coef. | Std. Err. | Coef. | Std. Err. |  |
| C D | .3256856 | .245626 | .4250335 | .166085 | -.0993479 | .2963072 | 0.737 |
| A C | -.7255493 | .1152472 | -1.03256 | .1466818 | .3070105 | .1866566 | 0.100 |
| A B | -.1808725 | .1216508 | .0235587 | .143628 | -.2044312 | .1882274 | 0.277 |
| A D | -.5525182 | .1918475 | -.3566903 | .1825452 | -.1958279 | .2648293 | 0.460 |
| B C | -.884404 | .1157063 | -.5280171 | .1470575 | -.3563869 | .1872988 | 0.057 |
| B D | -.1720106 | .2141785 | -.4737726 | .1732329 | .3017619 | .2755689 | 0.273 |

Note: A=Tuina, B=Acupuncture, C=Traction, D=Chinese herbs

5.3 VAS

| Side | Direct | | Indirect | | Difference | | P>\|z\| |
| --- | --- | --- | --- | --- | --- | --- | --- |
|  | Coef. | Std. Err. | Coef. | Std. Err. | Coef. | Std. Err. |  |
| C D | -1.01951 | .7251762 | -2.433964 | 1.019918 | 1.414455 | 1.251405 | 0.258 |
| A C | 1.085613 | .284755 | 1.860108 | .5386931 | -.7744952 | .6093627 | 0.204 |
| A B | .4367818 | .4412777 | -.3377266 | .42002 | .7745083 | .6093665 | 0.204 |
| B C | 1.533222 | .3079655 | .5175056 | .4648459 | 1.015717 | .5576999 | 0.069 |
| B D | -1.14 | .9814161 | .2744665 | .7764285 | -1.414466 | 1.251407 | 0.258 |

Note: A=Tuina, B=Acupuncture, C=Traction, D=Chinese herbs

5.4 JOA

| Side | Direct | | Indirect | | Difference | | P>\|z\| |
| --- | --- | --- | --- | --- | --- | --- | --- |
|  | Coef. | Std. Err. | Coef. | Std. Err. | Coef. | Std. Err. |  |
| C D | 5.559999 | 2.894943 | 7.449191 | 1116.307 | -1.889191 | 1116.311 | 0.999 |
| A C | -3.240477 | .9840358 | -5.831965 | 2.056468 | 2.591488 | 2.278695 | 0.255 |
| A B | -.859759 | 1.720269 | 1.731951 | 1.496703 | -2.59171 | 2.278747 | 0.255 |
| B C | -4.972409 | 1.127634 | -2.380739 | 1.980515 | -2.59167 | 2.278753 | 0.255 |

Note: A=Tuina, B=Acupuncture, C=Traction, D=Chinese herbs

## 6 Results of pair-wise meta-analyses

| Endpoints | Comparison | study number | I^2^ | Effects (MD/OR, 95%CI) |
| --- | --- | --- | --- | --- |
| Invalid rate | Traction vs Chinese herbs | 8 | 0% | 2.1 (1.30, 3.39) |
|  | Acupuncture vs Chinese herbs | 12 | 21.7% | 0.79 (0.57, 1.08) |
|  | Tuina vs Chinese herbs | 12 | 49.4% | 0.53 (0.39, 0.72) |
|  | Tuina vs Traction | 33 | 7.9% | 0.46 (0.38, 0.56) |
|  | Tuina vs Acupuncture | 29 | 39.1% | 0.95 (0.76, 1.17) |
|  | Acupuncture vs Traction | 27 | 0% | 0.32 (0.26, 0.39) |
| Cure rate | Traction vs Chinese herbs | 8 | 55.3% | 0.80 (0.59, 1.08) |
|  | Acupuncture vs Chinese herbs | 12 | 0% | 1.10 (0.83, 1.47) |
|  | Tuina vs Chinese herbs | 12 | 0% | 1.35 (1.08, 1.69) |
|  | Tuina vs Traction | 32 | 0% | 1.46 (1.28, 1.67) |
|  | Tuina vs Acupuncture | 30 | 0% | 1.09 (0.94, 1.25) |
|  | Acupuncture vs Traction | 27 | 1.9% | 1.54 (1.38,1.72) |
| VAS | Traction vs Chinese herbs | 2 | 89.1% | 0.97 (-0.00, 1.95) |
|  | Acupuncture vs Chinese herbs | 1 | N/A | 1.08 (0.57, 1.59) |
|  | Tuina vs Traction | 12 | 72.1% | -0.90 (-1.17, -0.64) |
|  | Tuina vs Acupuncture | 5 | 96.9% | -0.58 (-1.70, 0.54) |
|  | Acupuncture vs Traction | 9 | 97.7% | -3.10 (-4.23, -1.97) |
| JOA | Traction vs Chinese herbs | 1 | N/A | -3.15 (-3.77, -2.52) |
|  | Tuina vs Traction | 9 | 79.4% | 1.12 (0.79, 1.45) |
|  | Tuina vs Acupuncture | 3 | 98.3% | -0.55 (-2.51, 1.42) |
|  | Acupuncture vs Traction | 7 | 98.2% | 2.21 (0.67, 3.75) |

## 7 GRADE for outcome measurements

7.1 Invalid rate

7.1.1 Direct comparisons

|  | Study limitations | Imprecision | Inconsistency | Indirectness | Publication bias | GRADE |
| --- | --- | --- | --- | --- | --- | --- |
| Traction vs Chinese herbs | Downgrade | No downgrade | No downgrade | No downgrade | Downgrade | low |
| Acupuncture vs Chinese herbs | Downgrade | No downgrade | No downgrade | No downgrade | Downgrade | low |
| Tuina vs Chinese herbs | Downgrade | No downgrade | No downgrade | No downgrade | Downgrade | low |
| Tuina vs Traction | Downgrade | No downgrade | No downgrade | No downgrade | Downgrade | low |
| Tuina vs Acupuncture | Downgrade | No downgrade | No downgrade | No downgrade | Downgrade | low |
| Acupuncture vs Traction | Downgrade | No downgrade | No downgrade | No downgrade | Downgrade | low |

7.1.2 Indirect comparisons

|  | the best way of indirect comparison | GRADE |
| --- | --- | --- |
| Traction vs Chinese herbs | Acupuncture vs Traction, Acupuncture vs Chinese herbs | low |
| Acupuncture vs Chinese herbs | Acupuncture vs Traction, Traction vs Chinese herbs | low |
| Tuina vs Chinese herbs | Tuina vs Traction, Traction vs Chinese herbs | low |
| Tuina vs Traction | Tuina vs Acupuncture, Traction vs Acupuncture | low |
| Tuina vs Acupuncture | Tuina vs Traction, Traction vs Acupuncture | low |
| Acupuncture vs Traction | Acupuncture vs Tuina, Tuina vs Traction | low |

Summary

| Traction vs Chinese herbs | low |
| --- | --- |
| Acupuncture vs Chinese herbs | low |
| Tuina vs Chinese herbs | low |
| Tuina vs Traction | low |
| Tuina vs Acupuncture | low |
| Acupuncture vs Traction | low |

7.2 Cure rate

7.2.1 Direct comparison

|  | Study limitations | Imprecision | Inconsistency | Indirectness | Publication bias | GRADE |
| --- | --- | --- | --- | --- | --- | --- |
| Traction vs Chinese herbs | Downgrade | No downgrade | Downgrade | No downgrade | Downgrade | very low |
| Acupuncture vs Chinese herbs | Downgrade | No downgrade | No downgrade | No downgrade | Downgrade | low |
| Tuina vs Chinese herbs | Downgrade | No downgrade | No downgrade | No downgrade | Downgrade | low |
| Tuina vs Traction | Downgrade | No downgrade | No downgrade | No downgrade | Downgrade | low |
| Tuina vs Acupuncture | Downgrade | No downgrade | No downgrade | No downgrade | Downgrade | low |
| Acupuncture vs Traction | Downgrade | No downgrade | No downgrade | No downgrade | Downgrade | low |

7.2.2 Indirect comparisons

|  | the best way of indirect comparison | GRADE |
| --- | --- | --- |
| Traction vs Chinese herbs | Acupuncture vs Traction, Acupuncture vs Chinese herbs | low |
| Acupuncture vs Chinese herbs | Acupuncture vs Traction, Traction vs Chinese herbs | very low |
| Tuina vs Chinese herbs | Tuina vs Traction, Traction vs Chinese herbs | very low |
| Tuina vs Traction | Tuina vs Acupuncture, Traction vs Acupuncture | low |
| Tuina vs Acupuncture | Tuina vs Traction, Traction vs Acupuncture | low |
| Acupuncture vs Traction | Acupuncture vs Tuina, Tuina vs Traction | low |

7.2.3 Summary

| Traction vs Chinese herbs | low |
| --- | --- |
| Acupuncture vs Chinese herbs | low |
| Tuina vs Chinese herbs | low |
| Tuina vs Traction | low |
| Tuina vs Acupuncture | low |
| Acupuncture vs Traction | low |

7.3 VAS

7.3.1 Direct comparisons

|  | Study limitations | Imprecision | Inconsistency | Indirectness | Publication bias | GRADE |
| --- | --- | --- | --- | --- | --- | --- |
| Traction vs Chinese herbs | Downgrade | Downgrade | Downgrade | No downgrade | Downgrade | very low |
| Acupuncture vs Chinese herbs | Downgrade | Downgrade | No downgrade | No downgrade | Downgrade | very low |
| Tuina vs Traction | Downgrade | No downgrade | Downgrade | No downgrade | Downgrade | very low |
| Tuina vs Acupuncture | Downgrade | No downgrade | Downgrade | No downgrade | Downgrade | very low |
| Acupuncture vs Traction | Downgrade | No downgrade | Downgrade | No downgrade | Downgrade | very low |

7.3.2 Indirect comparisons

|  | the best way of indirect comparison | GRADE |
| --- | --- | --- |
| Traction vs Chinese herbs | Acupuncture vs Traction, Acupuncture vs Chinese herbs | very low |
| Acupuncture vs Chineseherbs | Acupuncture vs Traction, Traction vs Chinese herbs | very low |
| Tuina vs Chinese herbs | Tuina vs Traction, Traction vs Chinese herbs | very low |
| Tuina vs Traction | Tuina vs Acupuncture, Traction vs Acupuncture | very low |
| Tuina vs Acupuncture | Tuina vs Traction, Traction vs Acupuncture | very low |
| Acupuncture vs Traction | Acupuncture vs Tuina, Tuina vs Traction | very low |

7.3.3 Summary

| Traction vs Chinese herbs | very low |
| --- | --- |
| Acupuncture vs Chinese herbs | very low |
| Tuina vs Chinese herbs | very low |
| Tuina vs Traction | very low |
| Tuina vs Acupuncture | very low |
| Acupuncture vs Traction | very low |

7.4 JOA

7.4.1 Direct comparisons

|  | Study limitations | Imprecision | Inconsistency | Indirectness | Publication bias | GRADE |
| --- | --- | --- | --- | --- | --- | --- |
| Traction vs Chinese herbs | Downgrade | Downgrade | No Downgrade | No downgrade | Downgrade | very low |
| Tuina vs Traction | Downgrade | No downgrade | Downgrade | No downgrade | Downgrade | very low |
| Tuina vs Acupuncture | Downgrade | Downgrade | Downgrade | No downgrade | Downgrade | very low |
| Acupuncture vs Traction | Downgrade | No downgrade | Downgrade | No downgrade | Downgrade | very low |

7.4.2 Indirect comparisons

|  | the best way of indirect comparison | GRADE |
| --- | --- | --- |
| Traction vs Chinese herbs | N/A | N/A |
| Acupuncture vs Chinese herbs | Acupuncture vs Traction, Acupuncture vs Chinese herbs | very low |
| Tuina vs Chinese herbs | Tuina vs Traction, Traction vs Chinese herbs | very low |
| Tuina vs Traction | Tuina vs Acupuncture, Traction vs Acupuncture | very low |
| Tuina vs Acupuncture | Tuina vs Traction, Traction vs Acupuncture | very low |
| Acupuncture vs Traction | Acupuncture vs Tuina, Tuina vs Traction | very low |

7.4.3 Summary

| Traction vs Chinese herbs | very low |
| --- | --- |
| Acupuncture vs Chinese herbs | very low |
| Tuina vs Chinese herbs | very low |
| Tuina vs Traction | very low |
| Tuina vs Acupuncture | very low |
| Acupuncture vs Traction | very low |

## 8 SUCRA and probability plots

8.1 Invalid rate


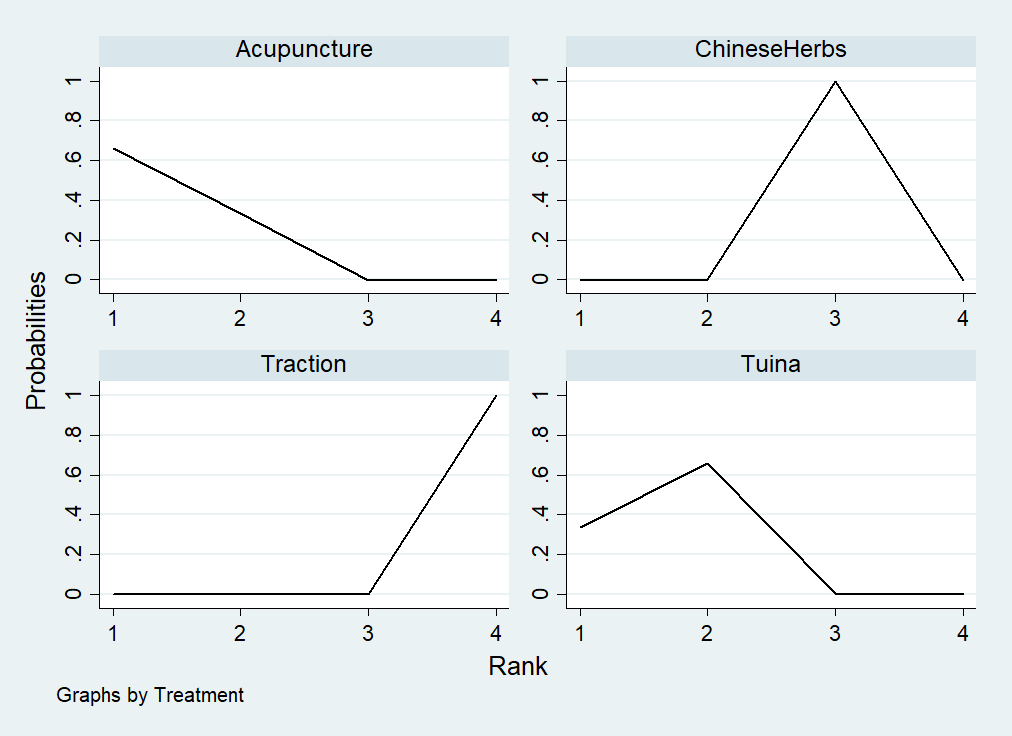


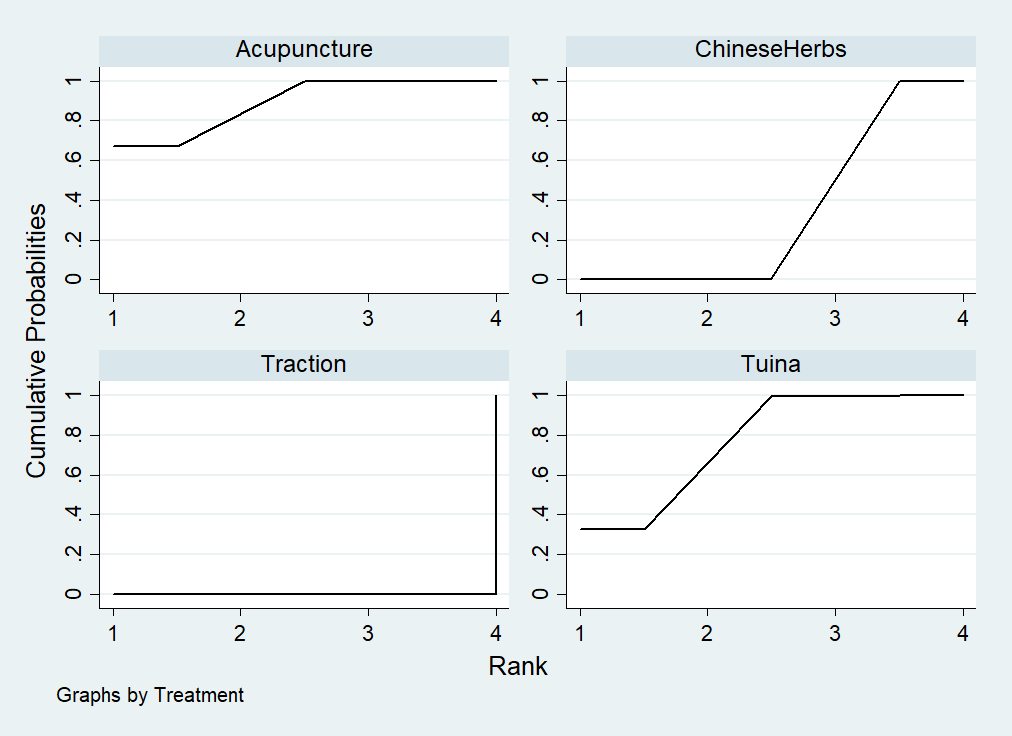


8.2 Cure rate


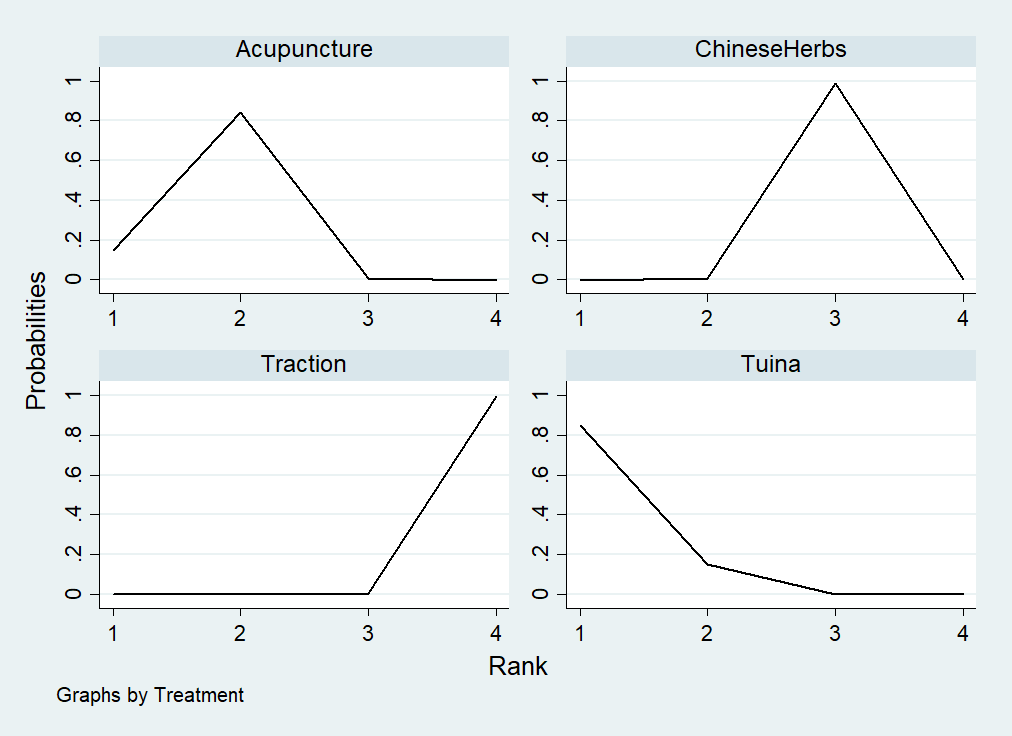


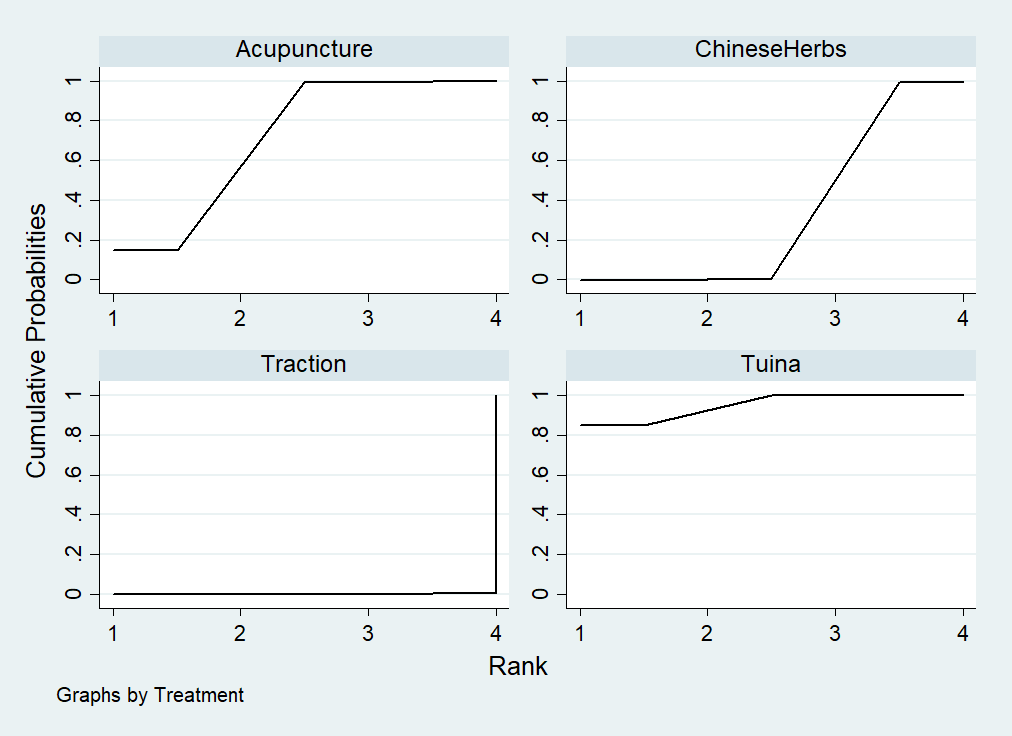


8.3 VAS


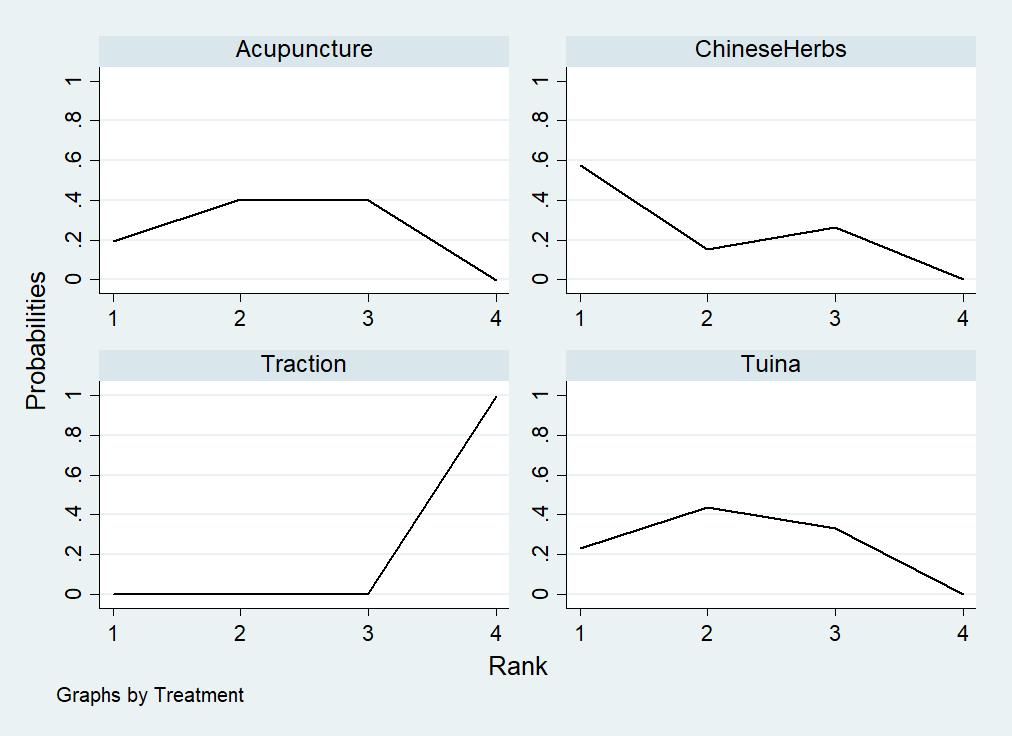


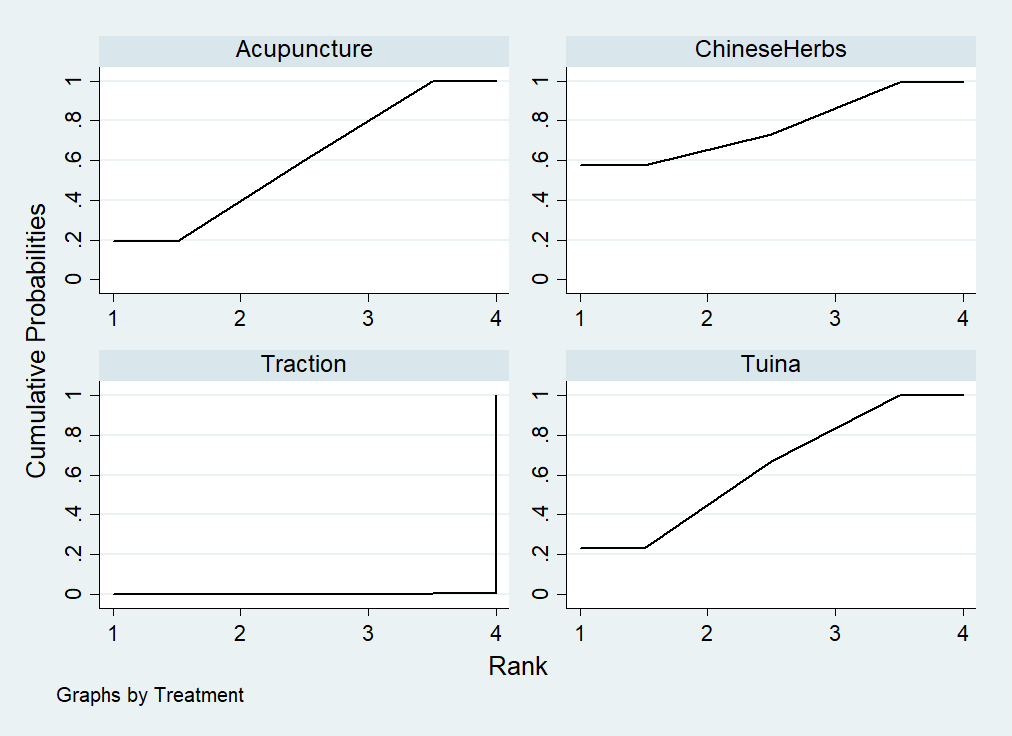


8.4 JOA


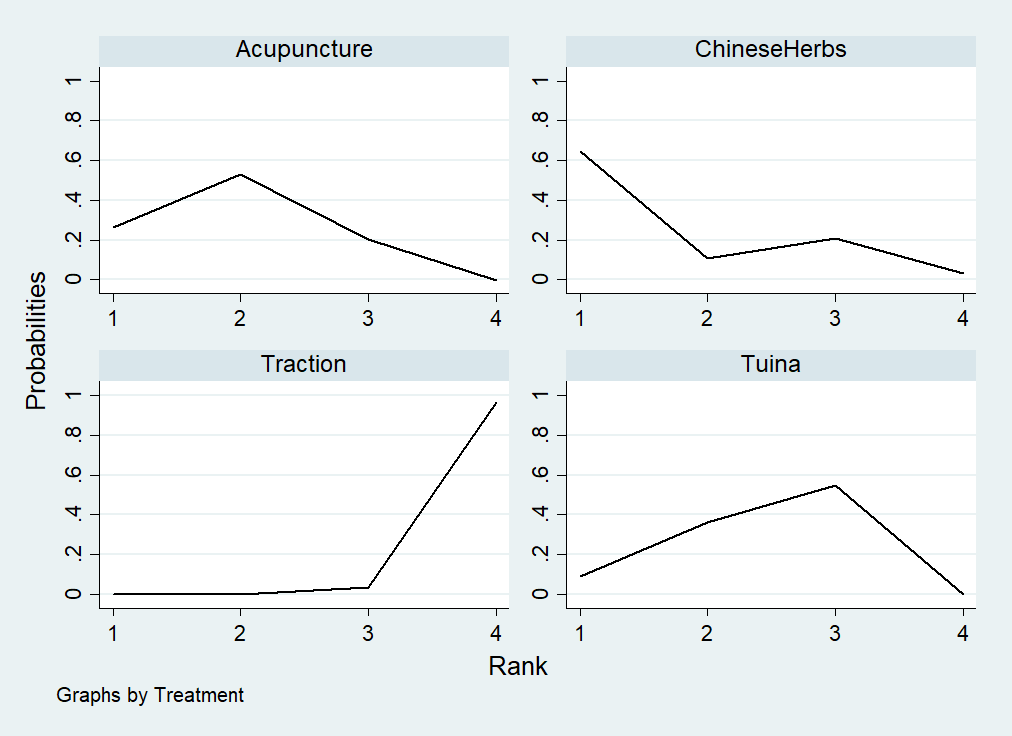


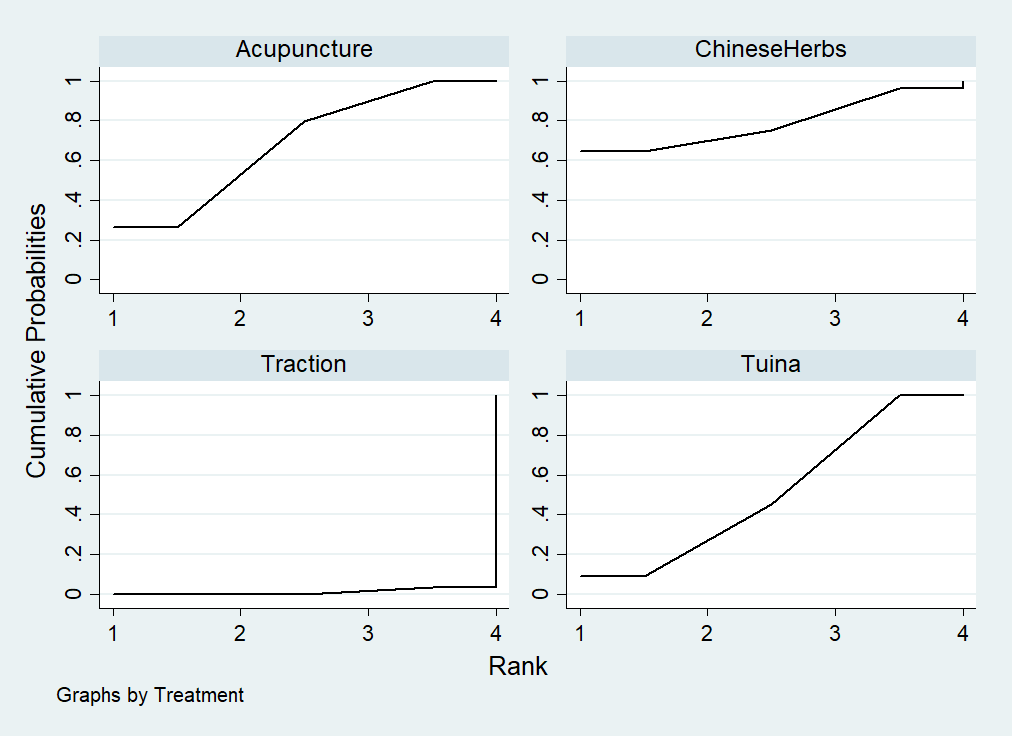

Supplement: Supplementary Materials — The supplementary appendix has 33 pages, including search strategy, references list of included studies, characteristics of included studies, risk of bias of included studies, node-splitting analysis, pair-wise meta-analysis, GRADE for outcome measurements, SUCRA, and probability plots. [file 6821310.f1.docx]
